# Identification of Mouse Claustral Neuron Types Based on Their Intrinsic Electrical Properties

**DOI:** 10.1523/ENEURO.0216-20.2020

**Published:** 2020-07-31

**Authors:** Martin Graf, Aditya Nair, Kelly L.L. Wong, Yanxia Tang, George J. Augustine

**Affiliations:** Lee Kong Chian School of Medicine, Nanyang Technological University, Singapore 308232

**Keywords:** neuron types, claustrum, electrophysiology, interneurons, projection neurons

## Abstract

Although its dense connections with other brain areas suggests that the claustrum is involved in higher-order brain functions, little is known about the properties of claustrum neurons. Using whole-cell patch clamp recordings in acute brain slices of mice, we characterized the intrinsic electrical properties of more than 300 claustral neurons and used unsupervised clustering of these properties to define distinct cell types. Differences in intrinsic properties permitted separation of interneurons (INs) from projection neurons (PNs). Five subtypes of PNs could be further identified by differences in their adaptation of action potential (AP) frequency and amplitude, as well as their AP firing variability. Injection of retrogradely transported fluorescent beads revealed that PN subtypes differed in their projection targets: one projected solely to subcortical areas while three out of the remaining four targeted cortical areas. INs expressing parvalbumin (PV), somatostatin (SST), or vasoactive intestinal peptide (VIP) formed a heterogenous group. PV-INs were readily distinguishable from VIP-INs and SST-INs, while the latter two were clustered together. To distinguish IN subtypes, an artificial neural network was trained to distinguish the properties of PV-INs, SST-INs, and VIP-INs, as independently identified through their expression of marker proteins. A user-friendly, machine-learning tool that uses intrinsic electrical properties to distinguish these eight different types of claustral cells was developed to facilitate implementation of our classification scheme. Systematic classification of claustrum neurons lays the foundation for future determinations of claustrum circuit function, which will advance our understanding of the role of the claustrum in brain function.

## Significance Statement

The function of the claustrum is mysterious. To better understand the claustrum, we examined the electrical properties of its neurons and identified eight neuron types. Differences in properties permitted clear separation of interneurons (INs) from projection neurons (PNs). PNs could be further subdivided based on differences in their physiological and anatomical characteristics. Although INs were heterogenous, a computational neural network could distinguish several subtypes. Our work is the first comprehensive analysis of claustrum neurons and provides important information about the physiological properties of these neurons. This work lays the foundation for advancing our understanding of signal processing within the claustrum and, thereby, elucidating how the claustrum contributes to brain information processing.

## Introduction

The claustrum is a thin sheet of gray matter positioned adjacent to the insular cortex and is the most densely interconnected region of the brain (Torgerson et al., 2015; Wang et al., 2017). Based on its rich neuronal connectivity, the claustrum has been proposed to play roles in higher cognitive functions such as consciousness (Crick and Koch, 2003, 2005), attention (Mathur, 2014; Remedios et al., 2014; Goll et al., 2015; Atlan et al., 2018; White et al., 2018), salience detection (Remedios et al., 2014), slow-wave sleep (Norimoto et al., 2020), or others (Smythies et al., 2012).

While the claustrum may be involved in higher cognitive functions, it remains entirely unclear how input is processed by different types of claustral neurons and their local circuits. To better understand claustral signal processing, it is fundamentally important to identify the types of neurons that exist within the claustrum. Previous work has described a few different types of claustral neurons in mice and rats (Shibuya and Yamamoto, 1998; Kim et al., 2016; Chia et al., 2017; White and Mathur, 2018) but did not comprehensively characterize claustrum neurons, because only a limited number of neurons and a limited set of characteristics were considered in each case. In particular, analysis of claustrum interneurons (INs) thus far has been limited to INs expressing the calcium-binding protein, parvalbumin (PV), and it is likely that several other types of INs also are present (Kowiański et al., 2001, 2009; Real et al., 2003; Kim et al., 2016; White and Mathur, 2018).

Here, we develop a comprehensive scheme for classifying claustral neurons. For this purpose, we measured the intrinsic electrical properties, anatomical projections and immunohistochemical properties of hundreds of claustrum neurons. We show that claustral projection neurons (PNs) can be clearly distinguished from INs based on their intrinsic electrical properties, and that both groups can be further divided into subtypes. PN were divided into five subtypes, with one of the groups exclusively projecting to the subcortex and several other subtypes projecting to the cortex. Claustral INs were also distinguished according to their expression of PV, somatostatin (SST), or vasoactive intestinal peptide (VIP). This classification scheme provides a firm cellular foundation for future studies that examine higher-order functions of the claustrum. We also developed software, based on artificial neuronal network algorithms, that identifies claustral neuron types based on their intrinsic electrical properties. This software provides an easy-to-use tool that will standardize claustral cell classification.

## Materials and Methods

### Animals

All animal experiments were approved by the Institutional Animal Care and Use Committee of Nanyang Technological University, Singapore. We used transgenic mice of both sexes from the following lines, all from The Jackson Laboratory: PV-cre (B6;129P2-*Pvalb^tm1(cre)Arbr^*/J; #008069), SST-cre (B6;129S4-*Sst^tm2.1(cre)Zjh^*/J; #013044), VIP-cre (B6:129S4-*Vip^tm1(cre)Zjh^*/J; #010908), choline acetyltransferase-cre (B6;129S6-*Chat^tm2(cre)Lowl^*/J; # 006410), and loxP-STOP-loxP-channelrhodopsin2-eYFP (B6;129S-Gt(ROSA)26Sor^tm32(CAG-COP4*H134R/EYFP)Hze^/J; #012569). The average age of mice used in our experiments was postnatal day 65.5 ± 0.6.

### Retrobead labeling of PNs

Retrograde transport of fluorescent latex microspheres (retrobeads) was used to identify PNs (Katz et al., 1984). To inject retrobeads into their brains, mice were anesthetized with isoflurane while kept on a heating blanket. A small craniotomy was performed using a dental drill. Hamilton syringes with a 33 gauge needle were used to inject either red (1:4 dilution in distilled water) or green (undiluted) retrobeads (Lumafluor) using the injection coordinates, angle, and volumes that are stated in Table 1. Injection speed was set to 10–15 nl/min. After the final volume was injected, the needles were kept in place for 5–10 min and then slowly retracted (1 mm/min). The wound was sutured, and acute brain slices were prepared from injected mice two to three weeks after surgery.

**Table 1:** Experimental details of the retrobead injections

Targetarea	Retrobeadspread into	Bregma(mm)	Dorso-ventral(mm)	Medial-lateral(mm)	Angle(°)	Lateral pialocation(mm)	Volume(nl)	Dilution	Backflowto
Cortex	VO/ LO	2.2	–2.3	1			250	1:4	
	Cg	1.34	–1.75	0.4			250	1:4	
		1.2	–1	0.3			250	Undiluted	
		1.1	–1.75	0.25			250	1:4	
	M1	1.35	–1	1.5			250	1:4	
	RS	–1.06	–1	0.25			150	1:4	
	mV1	–3.3	–0.4	2.1			250	Undiluted	
Subcortex	mHab; PV	–1.6	–2.4	0.25			150	Undiluted	
	CL, LPMR, MD (MDM,MDC, MDL)	–1.6	–3.25	0.45	14	1.25	150	Undiluted	Hab, Hip, MPtA
	CM, MD (MDC, MDL,MDM), PC, Rh, Sub	–1.7	–3.75	0.3	11	1	150	Undiluted	Hab, Hip, RS
	CM, MD (MDM, MDC),VM	–1.8	–3.3	0.3			100	Undiluted	Hab, Hip, PV
	CL, lHab, IMD, MD(MDM, MDC, MDL), VPPC	–1.94	–3.3	0.13	15	1	150	1:4	Hip, LPtA

Mice were injected into cortical or subcortical areas using the stated coordinates. Cg: cingulate cortex; CL: centrolateral thalamic nucleus; CM: Central medial thalamic nucleus; Hab: habenula; Hip: hippocampus; IMD: intermediodorsal thalamic nucleus; lHab: lateral habenula; LO: lateral orbital cortex; LPMR: lateral posterior thalamic nucleus; M1: primary motor cortex; MD: mediodorsal thalamus with subnuclei: MDC- Central, MDM: medial, MDL: lateral; mHab: medial habenular nucleus; MPtA: medial parietal association cortex; mV1: monocular primary visual cortex; PC: paracentral thalamic nucleus; PV: paraventricular thalamic nucleus; Rh: rhomboid thalamic nucleus; RS: retrosplenial cortex; Sub: submedius thalamic nucleus; VM: ventromedial thalamic nucleus; VPPC: parvicellular part of the ventral posterior nucleus of the thalamus.

### Brain slice experiments

Acute brain slices were prepared according to the general procedures described in Chia et al. (2017). Mice were deeply anesthetized with isoflurane and euthanized via decapitation. The brains were isolated and transferred into ice-cold sucrose solution containing the following: 250 mm sucrose, 26 mm NaHCO_3_, 10 mm glucose, 4 mm MgCl_2_, 3 mm myo-inositol, 2.5 mm KCl, 2 mm sodium pyruvate, 1.25 mm NaH_2_PO_4_, 0.5 mm ascorbic acid, 0.1 mm CaCl_2_, and 1 mm kynurenic acid, with an osmolality of 350–360 mOsm and a pH of 7.4. Coronal brain slices (250 to 300 μm thickness) were cut with a Leica VT 1000S vibratome. Slices were kept for 0.5 h at 34°C in artificial CSF (ACSF) containing the following: 126 mm NaCl, 24 mm NaHCO_3_, 1 mm NaH_2_PO_4_, 2.5 mm KCl, 2 mm CaCl_2_, 2 mm MgCl_2_, 10 mm glucose, and 0.4 mm ascorbic acid; 300–310 mOsm, pH 7.4, and gassed with a 95% O_2_/5% CO_2_ mixture before transfer to ACSF at room temperature for recordings.

### Identifying the claustrum in brain slices

**Figure 1.**
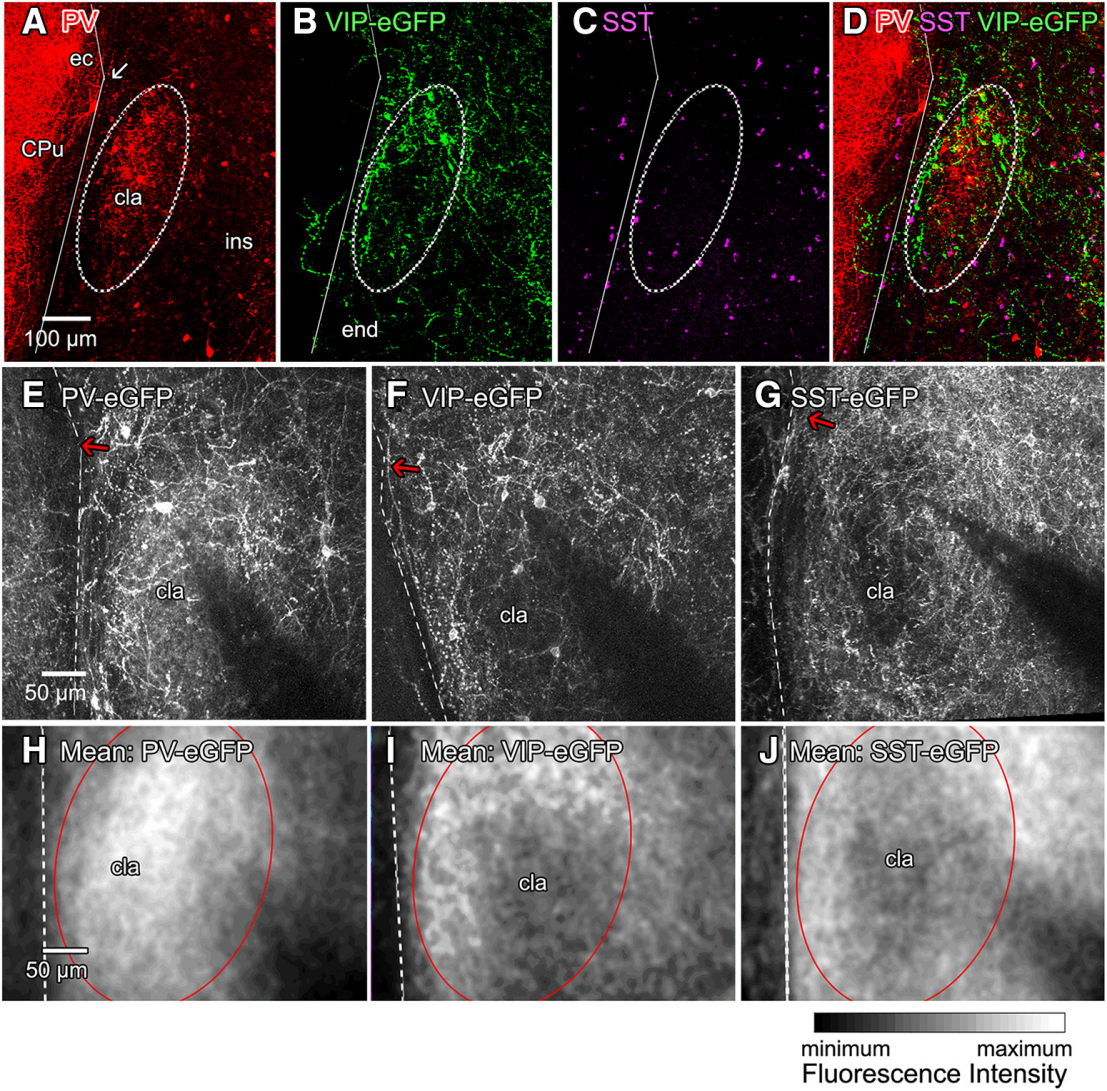
Identification of the claustrum in coronal brain slices. ***A***, The claustrum core (cla) is enriched in PV-positive neuropil and is positioned ventro-laterally relative to an inflection point of the external capsule (ec; arrow), between the caudate putamen (CPu) and insula (ins). The distribution of VIP-IN and SST-IN can also be used to identify the claustrum: both IN types differ in their neurite distribution between the PV-enriched claustrum core and its surrounding shell. ***B***, VIP-IN surround the claustrum core and their neurites form a mesh of neuropil around the PV-core. The neuropil density in insular cortex and endopiriform nucleus (end) is lower than in the claustral shell region. SST neurons (***C***) are enriched at the fringes of the claustrum core and avoid the PV-rich core. ***D***, Merger of A-C showing the PV-rich claustral core, embedded into the VIP-enriched neuropil of the claustral shell and an enrichment of SST neurons in the fringe area of the claustral core. ***E–G***, Visualization of IN-specific expression of eGFP in live brain slices from transgenic mice expressing eGFP in different populations of claustrum IN. Arrows indicate inflection points of the external capsule. ***E***, PV-promoter-driven eGFP expression is enriched in neuropil in the core of the claustrum. ***F***, VIP-promoter-driven eGFP expression is enriched in neuropil surrounding the claustrum core. ***G***, SST-promoter-driven eGFP expression shows that SST-positive neuropil has a complementary distribution to PV and is lowest in density in the claustrum core. ***H–J***, Images of mean intensity (gray scale) of eGFP fluorescence from 22–28 claustral image z-stacks from mice with eGFP expression in PV-IN (***H***), VIP-IN (***I***), and SST-IN (***J***). Images were aligned relative to the external capsule (dashed line), with the external capsule inflection point positioned at the upper left. Differences in fluorescence between the claustrum core (red outline) and its surrounds permit identification of the claustrum in live slices from all three mouse lines. The low fluorescence in the lower right corners of ***E–J*** is caused by glass recording pipettes.

Because the claustrum is a small and thin structure, it was important to have a reliable means of identifying it during brain slice recordings. The claustrum core is located ventro-laterally to an inflection point of the external capsule (Fig. 1*A*, arrow), which served as a reliable structural landmark. Further, PV, SST, and VIP were expressed in patterns that defined the location of the claustrum. PV was enriched in the claustral core (Fig. 1*A*,*D*), as reported previously (Real et al., 2003). VIP-INs formed a dense neurite plexus that partially overlapped with PV in the dorsal parts of the claustrum while ventral parts of the plexus were largely adjacent to the PV core (Fig. 1*B*,*D*). SST was enriched in the area directly adjacent to the PV core (Fig. 1*C*,*D*). This is the first demonstration that the position of VIP-IN or SST-IN can be used to locate the claustrum.

These IN-specific expression patterns served as the basis for locating claustral neurons during patch clamping in brain slices. Because our experiments used transgenic mouse lines with selective expression of membrane-bound eGFP in one of these three IN types, it was possible to guide electrode placement by using a two-photon microscope (Olympus FV-1000; 950-nm excitation wavelength) to visualize eGFP fluorescence in live slices. eGFP expression in PV-IN marked the claustrum core, while eGFP expression in VIP-IN or SST-IN could also be used to locate the claustrum because of the consistent distribution of the processes of these two subtypes of IN. This is illustrated for representative images of individual slices in Figure 1*E–G* and for images averaged across many slices in Figure 1*H–J*. VIP neurites formed a dense plexus at the height of the inflection point that corresponds to the most dorsal parts of the PV-enriched claustral core and were less prominent in the central core area of the claustrum (Fig. 1*F*,*I*). The distribution of SST neurons was complementary to that of the PV-enriched core, with a lower neurite density in an area ventral to the inflection point of the external capsule and within the claustrum core (Fig. 1*G*,*J*). Neurons were therefore considered to be within the claustrum if they were located within 50 μm of the PV-enriched or SST-deprived claustral core or within the dorsal part of the VIP-enriched neurite plexus or 200 μm ventral to this structure. Finally, in a majority of experiments, brain slices were fixed after completing recordings and then stained for PV expression and neurobiotin labeling to confirm the location of patched cells within the PV-enriched core of the claustrum. Taken together, we are confident that all results presented in this paper are based on recordings made from claustrum neurons.

### Patch clamp recordings of intrinsic electrical properties

The electrical properties of claustral neurons were measured with borosilicate glass pipettes (5–9 MΩ) filled with internal solution containing the following: 130 mm K-gluconate, 10 mm KOH, 2.5 mm MgCl_2_, 10 mm HEPES, 4 mm Na_2_ATP, 0.4 mm Na_3_GTP, 5 mm EGTA, 5 mm Na_2_ phosphocreatinin, and 0.2% neurobiotin (290–295 mOsm, pH 7.4). All whole-cell patch clamp recordings were performed at 24°C (except for a few recordings also done at 30°C) with a Multiclamp 700B amplifier (Molecular Devices) and a Digidata 1440 interface (Molecular Devices). Signals were acquired at 50 kHz and filtered at 10 kHz. The mean access resistance (R_a_) was 18.9 ± 0.4 MΩ (SEM) and remained non-compensated. Voltage values stated here also do not account for the liquid-liquid junction potential of −11.7 mV. Recordings were excluded from analysis if R_a_ exceeded 30 MΩ, the ratio of R_a_/R_m_ (R_m_ = membrane resistance) exceeded 20% and/or the resting membrane potential (RMP) was unstable or less negative than −50 mV. Action potentials (APs) also had to overshoot 0 mV to be included in our analysis. A few INs whose AP firing frequency did not saturate at the highest current intensity applied were also excluded because their maximum AP firing frequency and adaptation values could not be determined accurately.

**Figure 13.**
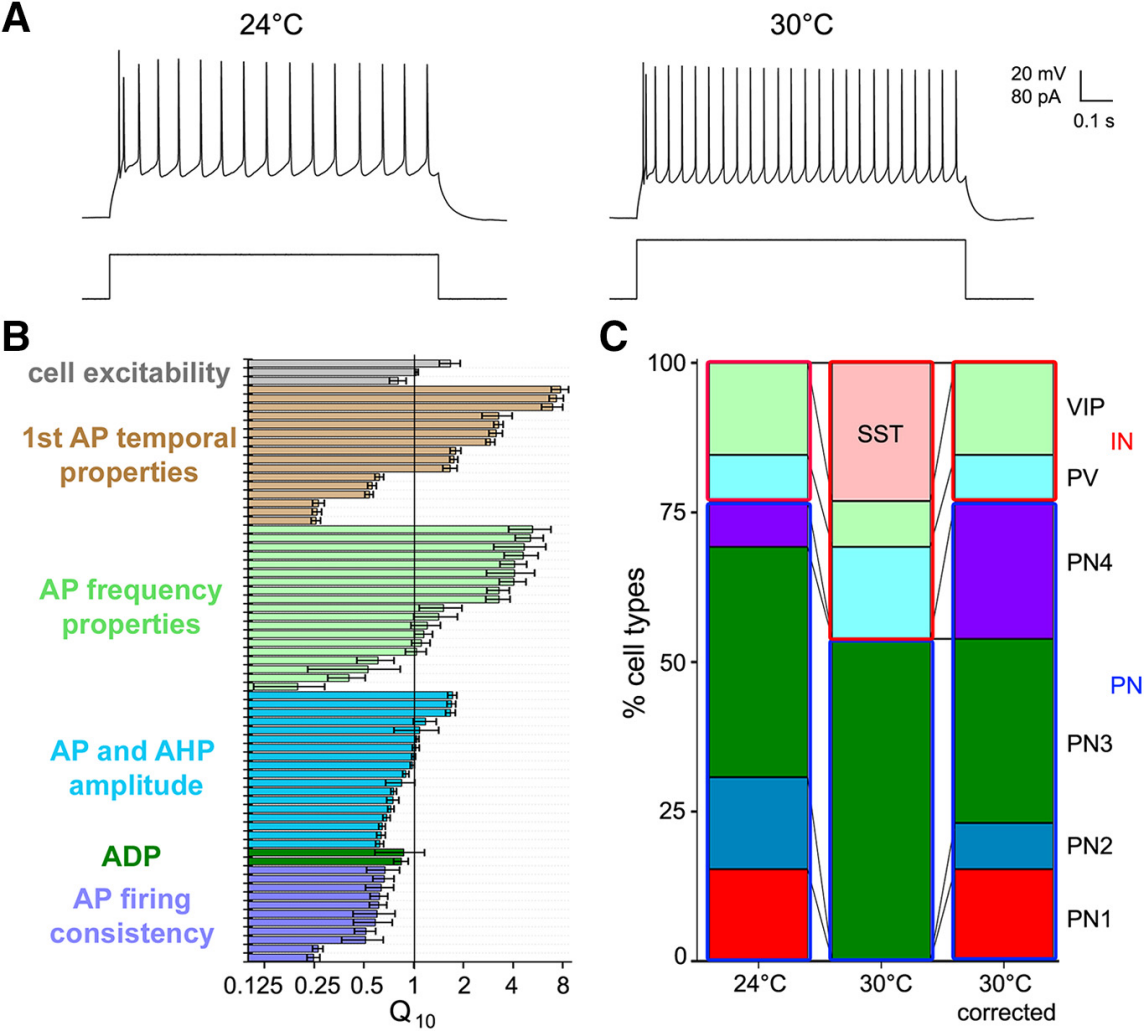
Effects of temperature on claustrum cell classification. ***A***, Representative example of the activity of a single PN3 neuron to a depolarizing current pulse at both 24°C (left) and at 30°C (right). ***B***, Relative changes in cell excitability (gray), first AP temporal properties (brown), AP frequency properties (pale green), AP and AHP amplitude (blue), ADP characteristics (green), and firing regularity (violet), expressed as Q_10_ values. Parameters are arranged as in Table 8, where precise Q_10_ values can be found. ***C***, Cell prediction results at 24°C (left) and 30°C, without (center) or with adjustment of the intrinsic cell features according to the Q_10_ values (right). Cell types are indicated on the right, IN are outlined by a red box, PN by a blue box. Adjustment of the intrinsic features by their mean Q_10_ values improved the prediction accuracy.

Electrical signals were analyzed with a variety of software: Clampfit 10.2 (Molecular Devices), Origin (OriginLab), Excel (Microsoft), Orange3 (Demsar et al., 2013), and custom-written MATLAB or R routines (MathWorks, R2015; R-Core Team). Up to 63 intrinsic electrical properties of claustrum neurons were measured in response to 1-s-long current pulses. RMP and R_m_ were derived from the slope of a linear fit of plots of the relationship between membrane potential and applied current, derived from a series of current steps ranging from −60 pA to the largest subthreshold depolarizing current. The current threshold (*ct*) was defined as the minimum current level required to elicit AP firing, with “ct*2” corresponding to twice the threshold current level and “max activity” corresponding to the depolarization level that evoked the highest AP frequency.

**Figure 2.**
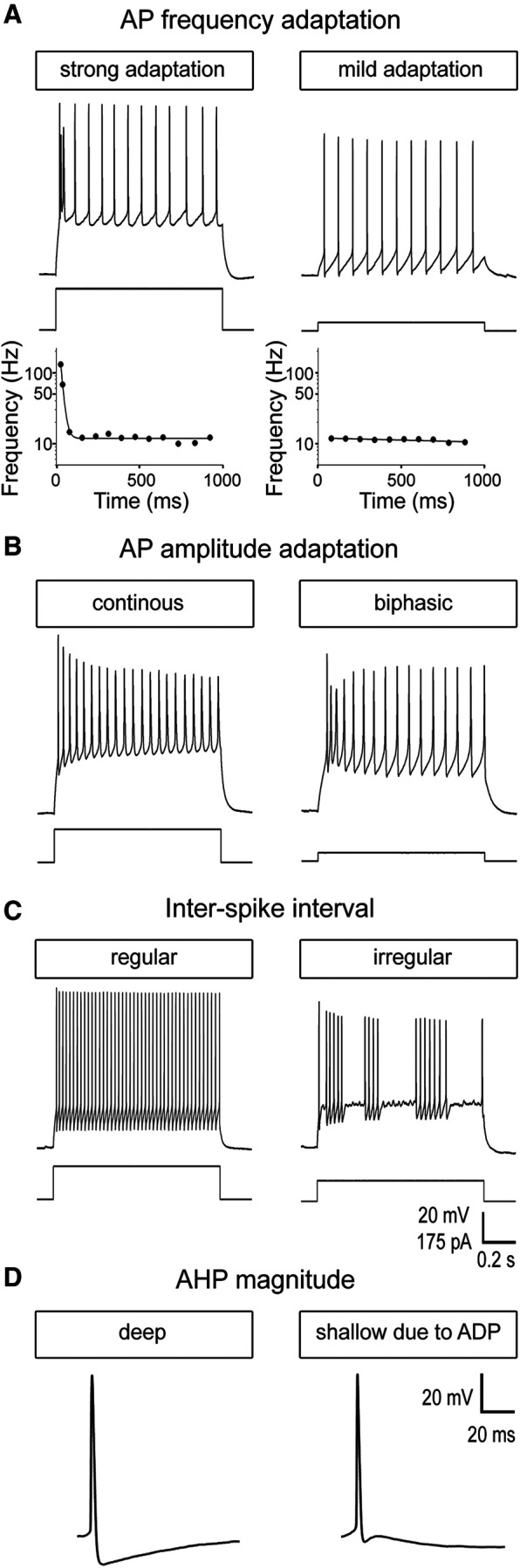
Claustral neurons are heterogenous in their responses to depolarizing currents. ***A***, top, Representative membrane potential responses to depolarizing current pulses show that AP firing patterns differed in their frequency adaptation properties. Bottom, Time course of AP frequency adaptation in the traces shown above. Further differences between claustral neurons were found for their AP amplitude adaptation (***B***), the ISI, (***C***) and the magnitude of their post-AP AHP (***D***).

AP threshold was defined as the membrane potential at which the first derivative of the membrane potential was >10 mV/ms. The threshold level was then used as a reference point to determine AP amplitude, half-width, threshold/AP amplitude, AP rise rate/half-width, and afterhyperpolarization (AHP) amplitude. AP waveform was described by the maximum AP rise and decay time as well as the ratio of maximum rise/decay time. Some parameters were the ratios of individual properties and were included to improve IN separation. The presence of an afterdepolarization (ADP; see Fig. 2*D* below) was measured at the *ct* level. If the AHP was followed by a local maximum, this indicated an ADP. To calculate integrated ADP amplitude, a linear fit of the membrane potential between the AHP and the local minimum after the ADP was subtracted from the trace and the resulting positive values were averaged. The local minimum was defined as the minimum value after the AHP that preceded the change from a negative to a positive value of the low-pass filtered (eight-pole Bessel, 50 Hz cutoff frequency) membrane potential slope.

The 63 intrinsic electrical properties also included many measurements of the temporal structure of repetitive AP firing. Interspike interval (ISI) was defined as the time between the peak overshoots of two consecutive APs, while the instantaneous frequency was the inverse of the ISI. AP frequency adaptation, which is a time-dependent reduction in AP frequency during the stimulus, was quantified as the difference between the instantaneous frequency of the first two APs and the mean instantaneous frequency of the last three APs, while relative AP adaptation (ISI ratio) was defined as the ratio of these two parameters. To characterize the tendency of some neurons to fire an initial burst of APs, we measured the initial AP instantaneous frequency, the mean and SD of the first two ISIs, as well as the change of the ISI interval (Toledo-Rodriguez et al., 2004). The “maximum initial adaptation change” describes the maximum difference of the initial instantaneous frequency for the first AP pair measured for two consecutive depolarization steps divided by the current difference between the two depolarizations. The “maximum initial adaptation change (*2)” describes the maximum initial adaptation change but ignores initial *ct* values and values following traces with only a single AP. “Peak adaptation level” is the current level at which the maximum initial adaptation change took place and is calculated relative to the *ct* value. AP firing variability (Cv2) within a spike train was calculated as the mean Cv2 values for all consecutive pairs of ISIs (Holt et al., 1996). To characterize firing variability after the first (Cv2-first AP) or the first two ISI pairs (Cv2-first/second AP), the initial one or two ISI values were excluded for the averaged Cv2 calculation. AP amplitude ratios for the “first/second,” “second/third,” and “first/last three AP” were derived from the absolute amplitude values of the corresponding APs.

### Measurement of temperature sensitivity

To determine the effect of temperature on the intrinsic electrical properties of claustrum neurons, these properties were recorded at both 24°C and 30°C for 13 neurons. The temperature coefficient, Q_10_ (see Eq. 1 below), was calculated for all properties in each individual neuron. From all Q_10_ values, the interquartile range (IQR) between the first (Q1) and the third (Q3) quartiles was calculated. Q_10_ values that were either smaller than Q1-3*IQR or larger than Q3+3*IQR were considered to be outliers and were discarded; means of the remaining Q_10_ values were used to correct parameters measured at 30°C to a temperature of 24°C.

### Cell clustering

To identify groups of cells that shared similar features, an unsupervised hierarchical clustering of intrinsic electrical property measurements was done with ClustVis software (Metsalu and Vilo, 2015). Raw data were scaled by the SD of population means for each parameter (Z-score). For the entire population, a list of 38 properties was used (Table 2), and neurons were clustered based on similarity of correlations between their features, with clustering distances between neurons calculated by the Pearson’s correlation. To separate neurons into distinct clusters with increasing dissimilarity, an average linkage criterion was used. To separate INs and PNs into distinct subclusters, Euclidean distances with Ward linkage were used. For IN subclustering, an extended list of 63 features was used. Dendrograms and Z-score maps were then generated in ClustVis according to similarities in their features, with the most similar cell pairs at the base of a branch. To identify an optimal number of cell clusters and validate the quality of the classification scheme, a silhouette analysis was performed and the average silhouette index (SI) was calculated using the package “cluster” in the R programming language (Maechler, 2018; R-Core-Team, 2018). The silhouette plot illustrates a measure, ranging from −1 to 1, of proximity between neighboring clusters; a width of 1 indicates that a cluster is highly separated from neighboring clusters, while a value of −1 indicates poor separation. The average silhouette width for all possible clusters was calculated and the optimal number of clusters was selected based on the maximum average width, which represents the optimal separation of clusters. As alternative clustering methods, t-distributed stochastic neighbor embedding (t-SNE; van der Maaten and Hinton, 2008) and/or locally linear embedding (LLE; Roweis and Saul, 2000) were applied using Orange3 (Demsar et al., 2013). All datasets were normalized, Euclidean distances were used as distance measure, data points were initialized with PCA, the learning rate was 200, and the number of maximum iterations was kept at 1000. Dataset-specific t-SNE settings were: “all neurons”: perplexity: 25, exaggeration: 1; “PN” set: perplexity: 8, exaggeration: 7; “IN” set: perplexity: 7, exaggeration: 7. The “PN” and “IN” set was additionally analyzed using LLE with the following settings: modified LLE, number of neighbors: IN: 13; PN: 14, max number of iterations 100.

**Table 2:** Subpopulation means and medians of claustral INs and PNs

Group		Interneurons				Projection neurons				Statisticalcomparison	
#	Property	Mean	SEM	Median	MAD	Mean	SEM	Median	MAD	*p* value	Mann–Whitney *U*
1	RMP (mV)	–63.3	0.45	–63.7	4.00	–71.9	0.26	–72.0	2.28	5.8E-36	2.6E+03
2	Rm (MΩ)	389	19.9	326	138	246	9.22	227	68.8	4.2E-09	8.2E+03
3	ct	87.4	5.95	60.0	40.0	136	5.84	120	40.0	8.2E-11	7.7E+03
4	ADP probability	0.13	0.03	0.00	0.00	0.62	0.04	1.00	0.00	2.1E-19	6.8E+03
5	Mean ADP value (mV)	0.13	0.04	0.00	0.00	0.87	0.08	0.54	0.54	2.1E-19	6.5E+03
6	Max initial adaptation change (Hz/pA)	1.51	0.08	1.36	0.66	1.68	0.09	1.41	0.77	3.1E-01	1.2E+04
7	Max adaptation change relative to ct	2.01	0.13	1.33	0.33	1.60	0.04	1.43	0.24	3.4E-01	1.2E+04
8	Initial adaptation change (2) (Hz/pA)	1.05	0.07	0.71	0.23	1.49	0.08	1.17	0.57	3.0E-06	9.3E+03
9	Max adaptation change relative to ct (2)	2.42	0.13	2.00	0.70	1.65	0.04	1.50	0.25	3.7E-06	9.3E+03
10	AHP amplitude (mV)	–19.3	0.45	–19.1	4.26	–12.5	0.27	–12.0	2.58	1.3E-26	4.2E+03
11	AHP latency (ms)	6.25	0.88	3.60	1.10	42.8	4.26	11.3	7.43	1.3E-32	3.1E+03
12	Max AP rise (mV/ms)	187	4.61	180	39.1	194	3.08	192	30.5	7.1E-02	1.2E+04
13	Max AP decay (mV/ms)	–74.0	3.14	–61.7	19.5	–38.7	0.51	–37.5	4.69	3.7E-28	3.9E+03
14	AP half-width (ms)	1.26	0.04	1.22	0.37	1.84	0.03	1.78	0.19	4.5E-26	4.3E+03
15	AP threshold (mV)	–34.3	0.35	–34.5	2.98	–33.8	0.22	–34.0	2.02	2.3E-01	1.2E+04
16	AP amplitude (mV)	68.9	0.66	69.7	5.58	75.6	0.38	76.2	3.16	1.2E-14	6.7E+03
17	2xct: number of AP	33.5	2.15	22.0	11.0	15.5	0.38	15.0	4.0	7.4E-09	8.3E+03
18	2xct: latency to first AP	25.0	1.74	18.3	10.6	21.9	0.67	20.6	4.85	1.8E-01	1.2E+04
19	2xct: AHP amplitude (mV)	–15.8	0.41	–15.5	3.59	–5.98	0.33	–5.63	2.80	8.9E-40	2.0E+03
20	2xct: AHP latency (ms)	3.35	0.14	3.10	0.91	4.03	0.05	4.04	0.44	1.2E-12	7.2E+03
21	Max AP rise (mV/ms)	192	4.93	185	37.5	195	3.44	187	31.8	2.8E-01	1.2E+04
22	Max AP decay (mV/ms)	–75.6	2.99	–62.5	18.8	–43.2	0.59	–42.2	4.69	1.8E-25	4.4E+03
23	2xct: AP half-width (ms)	1.21	0.03	1.17	0.31	1.69	0.02	1.67	0.14	2.7E-25	4.4E+03
24	2xct: AP threshold (mV)	–36.4	0.39	–36.2	3.02	–35.9	0.26	–36.1	2.24	4.0E-01	1.3E+04
25	2xct: AP amplitude (mV)	69.7	0.57	70.3	4.53	74.4	0.35	74.9	2.85	1.8E-10	7.8E+03
26	2xct: 1st/2nd AP ratio	1.08	0.01	1.06	0.03	1.33	0.01	1.31	0.13	1.5E-41	1.8E+03
27	2xct: 2nd/3rd AP ratio	1.02	0.00	1.02	0.01	0.95	0.01	0.96	0.07	3.3E-13	7.0E+03
28	2xct: 1st /last AP ratio	1.18	0.01	1.14	0.06	1.29	0.02	1.22	0.10	9.0E-08	8.7E+03
29	2xct: initial instant frequency	51.1	2.75	39.0	19.9	67.4	2.25	68.1	24.1	4.9E-07	9.0E+03
30	2xct: max adaptation (Hz)	17.1	1.10	14.7	9.00	54.3	2.24	55.9	23.8	3.2E-29	3.7E+03
31	2xct: ISI ratio	0.67	0.03	0.67	0.13	0.24	0.01	0.18	0.06	4.4E-39	2.1E+03
32	2xct: mean of first 2 ISI (ms)	32.9	1.82	29.3	14.7	29.9	1.34	26.9	9.19	7.3E-01	1.3E+04
33	2xct: SD of first 2 ISI (ms)	5.14	0.62	2.32	1.62	15.0	1.06	9.48	4.90	6.1E-25	4.5E+03
34	2xct: change of ISI duration from 1stto 2nd AP (ms)	3.09	1.02	2.61	2.01	21.2	1.50	13.4	6.93	2.1E-31	3.3E+03
35	2xct: change of instant frequencyfrom 1st to 2nd AP pair (Hz)	–6.42	0.71	–6.05	4.72	–34.1	1.57	–32.8	17.5	9.4E-35	2.8E+03
36	2xct: Cv2:all	0.12	0.01	0.07	0.03	0.21	0.01	0.18	0.07	4.4E-18	5.9E+03
37	2xct: Cv2-1st AP	0.11	0.01	0.06	0.03	0.16	0.01	0.12	0.05	7.4E-13	7.1E+03
38	2xct: Cv2-1st/2nd AP	0.11	0.01	0.06	0.03	0.14	0.01	0.10	0.04	8.3E-09	8.3E+03

Medians are indicated with their SEM, while medians are indicated with their median absolute deviation (MAD).

**Figure 12.**
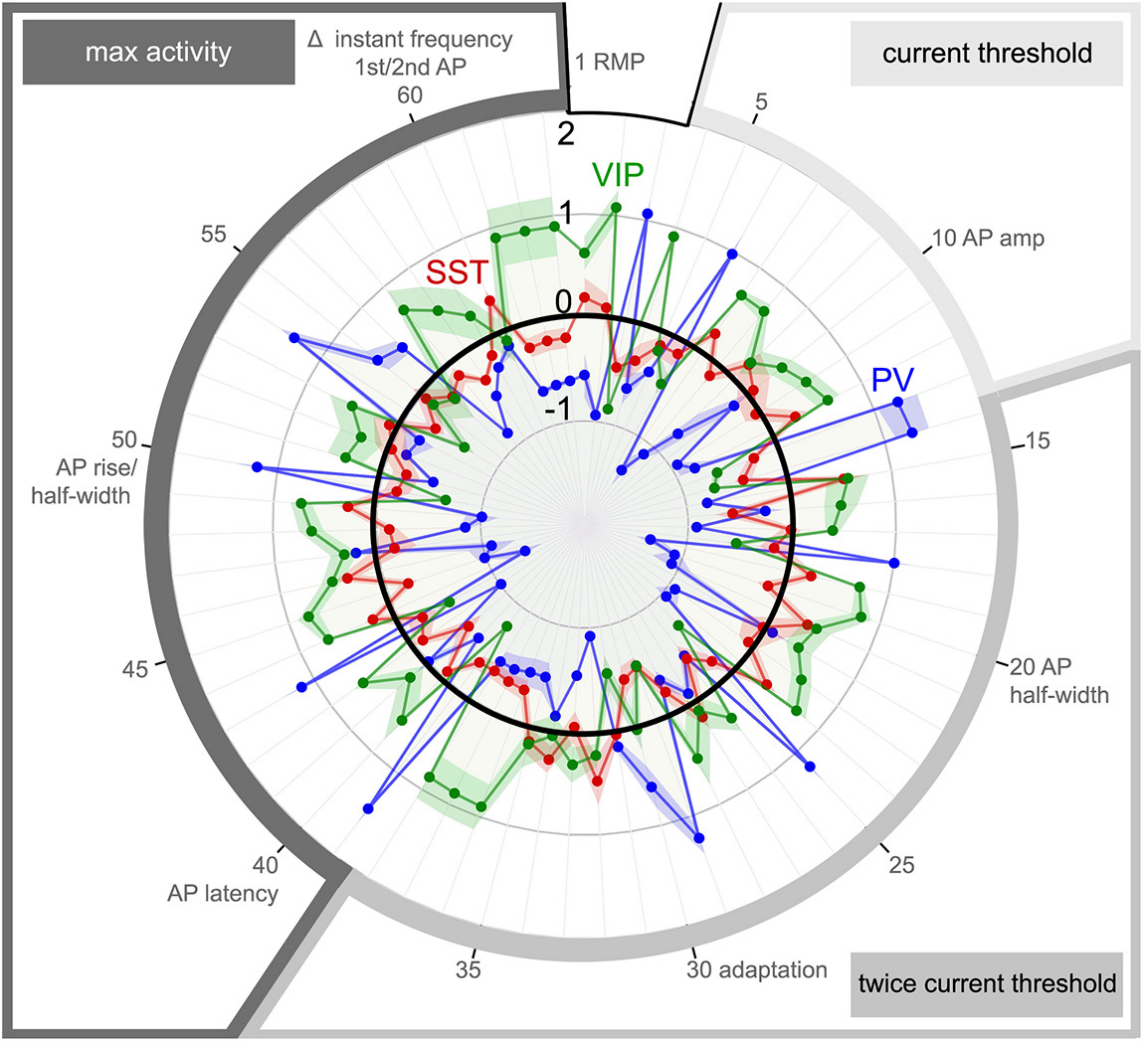
Properties of the three claustrum IN subtypes. Claustrum IN intrinsic electrical properties (expressed as Z-scores) measured in response to currents at the current threshold (light gray), at twice the current threshold (medium gray) and at the current eliciting maximal AP firing (max activity, dark gray). PV-IN properties are shown in blue, SST-IN in red, and VIP-IN in green. Values are means of subpopulation Z-scores, with shading indicating ±SEM, and black ring indicating IN population means. All 63 properties are numbered and arranged in the order shown in Table 6. The identity of every tenth property is also indicated.

### Immunohistochemistry

To visualize the location and morphology of patched neurons, as well as their expression of marker genes, brain slices were fixed in 4% paraformaldehyde overnight at 4°C and standard immunohistochemical processing procedures were followed. In short, brain slices were washed in PBS with 0.25% Triton X-100 (PBSTx) and incubated with primary antibody (rabbit-anti-PV, 1:1000, Swant: PV-28, RRID:AB_2315235) and/or rat-anti-SST (1:500, EMD Millipore: Mab354, RRID:AB_2255365), and/or chicken-anti-GFP (1:1000, Abcam: ab13970, RRID:AB_300798) and 1:1000 fluorophore-conjugated streptavidin (Alexa Fluor 405, Alexa Fluor 488, or Alexa Fluor 647; Thermo Fisher Scientific) for 48 h at 4°C. Brain slices were washed, incubated for 2 h at room temperature in fluorophore-conjugated secondary antibody (1:500, Alexa Fluor 488, Alexa Fluor 546, or Alexa Fluor 633; RRID:AB_142924, RRID:AB_2534093, RRID:AB_141778, Thermo Fisher Scientific), rinsed in PBSTx, and mounted in ProlongG-antifade reagent (Thermo Fisher Scientific).

### Image acquisition and processing

Images were acquired on an Olympus FV-1000 two-photon microscope. Image analysis and processing were done with Fiji/ImageJ software (Schindelin et al., 2012). As necessary, background subtraction (rolling ball radius 50 pixels) and/or a 5 × 5 convolved kernel filter (center: 24; remaining: −1) were used to optimize image signal/noise ratio.

### Machine-learning assisted automated classification of cell types

Because our classification scheme relies on combinations of multiple intrinsic electrical properties, we have produced an automated, user-friendly method to implement our classification scheme. We created a GUI-based web tool written in the R programming language and Shiny GUI framework (R-Core-Team, 2018) that classifies cell types based on the electrical properties described here.

The software consists of trained classifiers capable of distinguishing claustral cell types using feedforward neural networks with a single hidden layer. PCA was used to reduce the dimensionality of input data, and 27 components that captured 99% of variance in the data were used to train the network. Ten-fold cross validation was used for all three classification steps to choose the appropriate L2 regularization parameter and number of hidden neurons over a grid search of hyperparameters. The final model consisted of 10 hidden neurons and a weight decay parameter of 0.001 and achieved out-of-sample accuracy of 96%. The above was implemented using the caret package in the R language (Kuhn, 2008). Fourteen electrophysiological properties described in this paper are extracted from traces automatically and classifiers trained according to the scheme described in this paper are used to distinguish five different subtypes of claustral PNs or three different subtypes of claustrum INs. While classification of PN subtypes is based on the unsupervised scheme described here, classification of INs is based on a database consisting of confirmed IN-marker-expressing neurons (19 confirmed PV-INs, 28 SST-INs, and 30 VIP-INs).

### Software and code accessibility

This software is available as a web-based application via https://claustrum.shinyapps.io/online/ or can be downloaded from GitHub via https://github.com/adityanairneuro/claustrum.

### Statistical analysis

Mean values of intrinsic electrical properties measured in different neuronal types were compared using an unpaired *t*-test with Welch’s correction, if these properties were normally distributed. If the properties were not normally distributed, the medians of cell properties of different neuronal types were compared using a Mann–Whitney *U* test for two separate neuronal groups or a Kruskal–Wallis test and Dunn’s multiple comparison test when more than two groups were compared. To estimate the effect size of non-normally distributed parameters between groups, η^2^ was calculated (Tomczak and Tomczak, 2014). To compare more than two groups with normally distributed parameters, an ANOVA test with Tukey’s *post hoc t*-test was used. To estimate the effect sizes for between-groups comparisons of normally distributed parameters, *R*^2^ was used.

## Results

### Claustral neurons differ widely in their intrinsic electrical properties

To distinguish neuron types, we measured the intrinsic electrical properties of a total of 326 claustral neurons in brain slices prepared from 106 mice. All patched neurons were unambiguously located within the claustrum, identified with the procedures described in Materials and Methods. As shown in Figure 2, claustral neurons differed widely in their responses to prolonged (1 s) depolarizing current pulses. Noticeable differences were found in the amount of AP frequency adaptation (Fig. 2*A*), the adaptation of AP amplitude (Fig. 2*B*), the ISI (Fig. 2*C*), as well as the characteristics of AP repolarization (Fig. 2*D*). This diversity of intrinsic electrical properties indicates multiple types of claustrum neurons.

**Figure 3.**
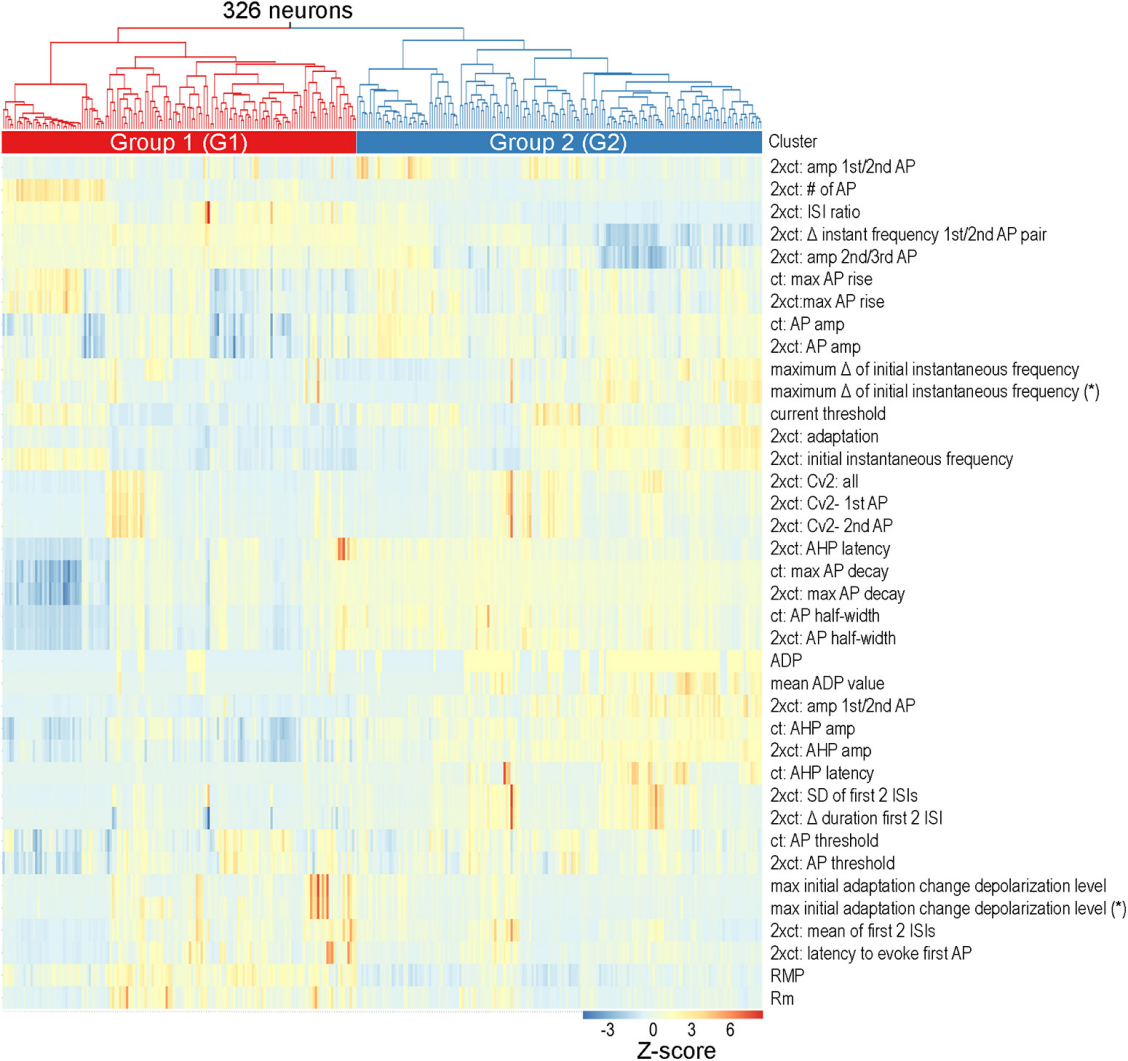
Unsupervised clustering of claustral neurons based on their intrinsic electrical properties. Top, Properties of 326 claustrum neurons were used to generate a dendrogram. Single cells are located at the lowest branches, with more similar cells clustered in closer proximity. At the highest level of classification, cells were split into two main groups (red and blue). Bottom, Z-scores of extracted intrinsic properties (rows) are shown for each individual cell (column). Hot colors indicate high Z-scores, while cool colors indicate low Z-scores.

**Figure 4.**
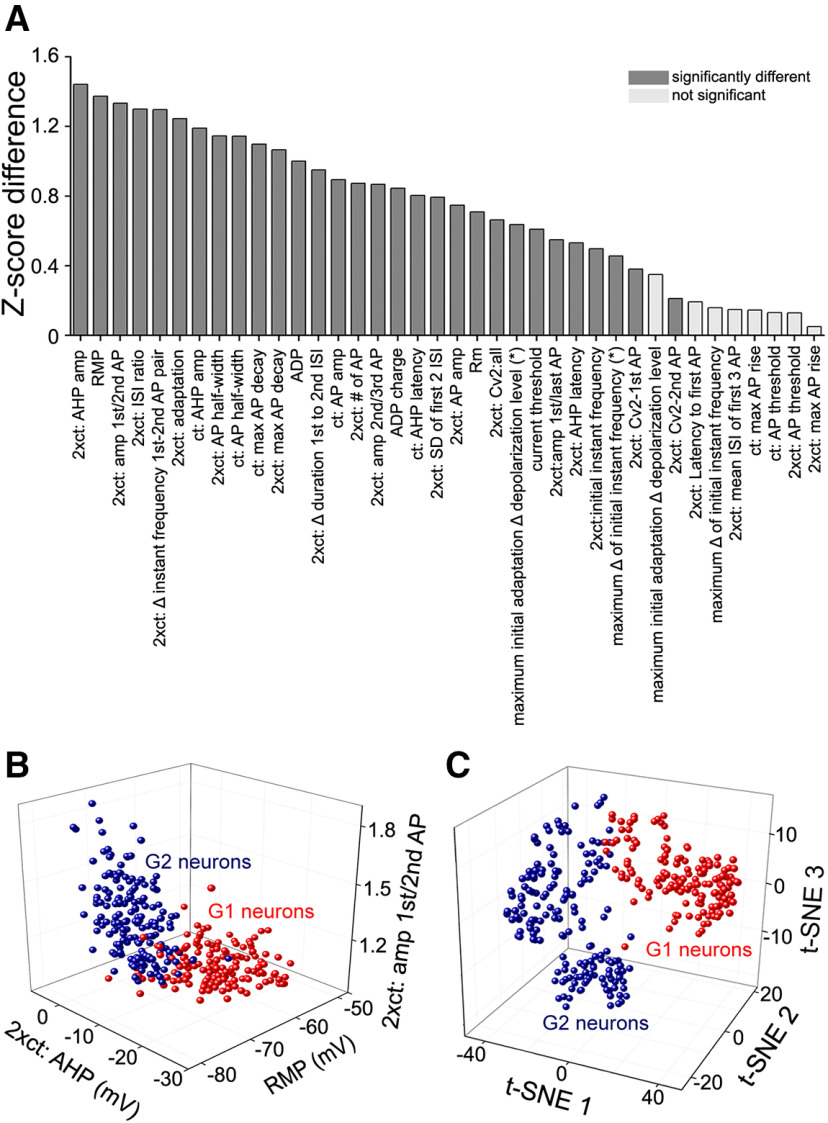
Differences between group 1 and group 2 claustrum neurons. ***A***, Ranking of absolute differences between the subpopulation Z-score means of G1 and G2 neurons. Largest differences were observed for AHP amplitude at *ct*x2, RMP and ratio of amplitudes of first and second APs at *ct*x2. Dark gray shading indicates medians that differed significantly between both groups, while light gray indicates non-significant differences; 30 out of 38 features showed a significant difference. ***B***, Comparison of the RMP, ratio of first/second AP amplitudes, and AHP amplitude at *ct*x2 for G1 and G2 neurons. Both groups were clearly separate, with the axis of the G1 cluster orthogonal to the G2 cluster axis. ***C***, Comparison of the intrinsic properties of G1 and G2 neurons, using t-SNE-based clustering. These groups did not overlap in the t-SNE feature space.

To identify subpopulations of claustrum neurons, 38 different intrinsic electrical properties were extracted from neuronal responses to depolarizing and hyperpolarizing current pulses (see Materials and Methods). These parameters were then normalized by their Z-scores and grouped using unsupervised agglomerative hierarchical clustering (Metsalu and Vilo, 2015). Claustral neurons (Fig. 3, columns) differed in their intrinsic properties (Fig. 3, rows) over a wide range, as indicated by the color-coded Z-scores. Cells with similar properties (similar colors) were clustered into groups, with more similar groups clustered into branches, and branches then clustered into subgroups according to their similarity, yielding the dendrogram shown in Figure 3, top. At the top hierarchical level, two main clusters of neurons could be distinguished: group 1 (G1; *n* = 152, 46.6%) and group 2 (G2; *n* = 174, 53.4%). These two clusters exhibited significant differences in their population medians for 30 out of 38 intrinsic properties (Table 2). To identify properties that differed the most between the groups, the absolute difference in the subpopulation Z-score means were calculated and ranked. The largest group differences in Z-score means for G1 and G2 were found for AHP magnitude at ct*2 and the RMP (Fig. 4*A*; Table 2), while the smallest differences were found for the max AP rise rate at ct*2 and AP threshold at ct*2 (Fig. 4*A*; Table 2). Plots of the three most divergent features revealed two distinct clusters that correlated well with G1 or G2 (Fig. 4*B*). As an alternative approach, neurons were also clustered using t-SNE (see Materials and Methods). Similar to the results of hierarchical clustering, G1 neurons clustered separately from G2 neurons (Fig. 4*C*). Taken together, the significant differences observed for most of the intrinsic electrical properties indicate that the two groups represent two different types of claustrum neurons.

### Identification of PNs and INs

**Figure 5.**
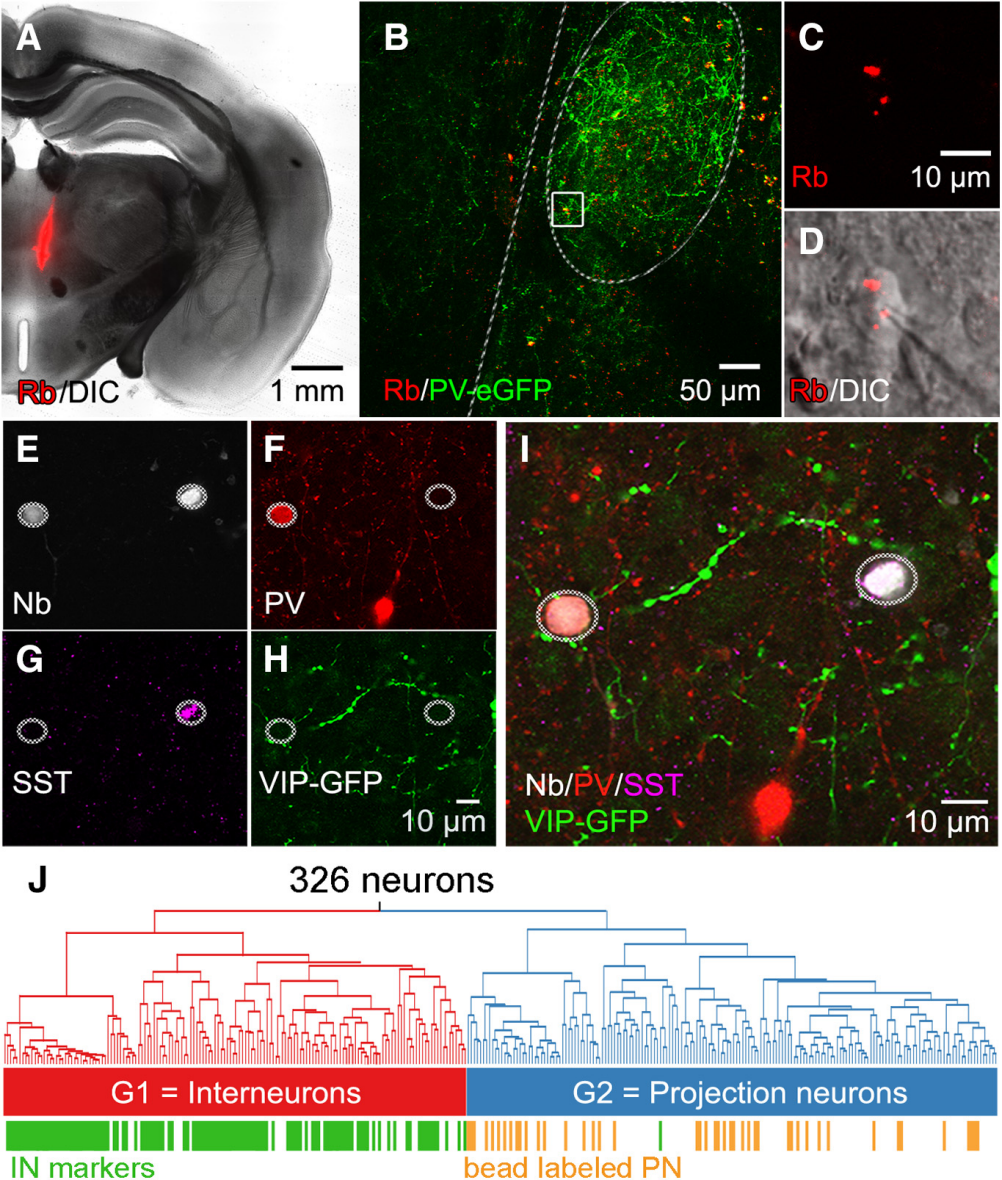
Identification of claustrum PNs and INs. Retrobead (Rb) labeling was used to identify PN, while marker protein expression was used to identify IN. ***A***, Overview of a Rb injection (pseudocolored red) targeting ventral and medial thalamic nuclei. ***B***, Coronal brain slice from the same animal in ***A***. The claustrum (dashed outline) can be identified by strong PV-eGFP expression (green), while subcortical-projecting neurons are labeled in red. ***C***, High-magnification image of Rb-label (pseudocolored red) in the subcortical-projecting claustrum neuron shown within the white square in panel ***B***. ***D***, Combined Rb and DIC image showing the same Rb-labeled neuron and the glass recording electrode. ***E***, Ovals indicate two neurobiotin-labeled claustral neurons. The left neuron expressed PV (***F***), while the right neuron expressed SST (***G***), and neither neuron expressed VIP-promoter-driven eGFP (***H***). ***I***, Merger of ***E–H*** indicates that the left cell is a PV-IN, while the cell on the right is an SST-IN. ***J***, Dendrogram from Figure 3, annotated with identified PN that were labeled with Rb (orange) and IN that expressed PV, SST, or VIP (green). IN markers were almost exclusively restricted to cells in the left arm of the dendrogram, identifying G1 as IN, while retrobead-labeled neurons were exclusively in the right arm of the dendrogram containing virtually no neurons expressing IN markers, indicating that G2 cells are PN.

In many brain regions, PNs and INs differ in their intrinsic electrical properties (McCormick et al., 1985). We next asked whether such differences underlie the two different groups of claustrum neurons. To identify PNs, fluorescent beads were injected into cortical areas (prefrontal, orbitofrontal, auditory, visual, motor cortex) or the subcortex (thalamus, habenula, hippocampus; Fig. 5*A*) of mice where the claustrum core could be identified by eGFP labeling (PV-cre × ChR2-eGFP; Fig. 5*B*). The cell bodies of PNs that retrogradely transported these beads could be visualized in live brain slices (Kim et al., 2016; Chia et al., 2017) and were located within the PV-enriched claustral core (Fig. 5*B–D*). Whole-cell patch clamp recordings were then used to determine the electrical properties of 44 of these labeled PNs. Remarkably, all PNs had G2 characteristics (Fig. 5*J*, orange), while G1 neurons were not labeled.

Because G1 cells did not appear to be PN, we determined whether these cells were INs by examining their expression of IN marker genes. The electrophysiological criteria described above were used to identify G1 cells, while the presence of IN marker genes was detected via two strategies. First, the fluorescence of eGFP expressed behind various IN promoters could be imaged in live slices to target IN for patch clamp recordings (Fig. 1*E–G*, *N* = 92). In addition, *post hoc* immunohistochemical (IHC) staining was used in patched cells that were filled with neurobiotin (Fig. 5*E*) to assess their expression of PV (Fig. 5*F*), SST (Fig. 5*G*), and/or VIP-promoter-driven eGFP (Fig. 5*H*,*I*). Neurons that expressed a specific IN marker were almost exclusively G1 cells (116/117 cells; 99.2%), with only a single VIP-expressing IN exhibiting G2 properties. Considering all neurobiotin-labeled G1 neurons, 87.9% (116/132) expressed IN markers (Fig. 5*J*, green). This is an underestimate, because not every cell was interrogated for all three IN markers. In contrast, only 1/95 G2 cells (1.1%; Fig. 5*J*) that were subjected to IHC expressed an IN marker (VIP), and every retrobead-labeled neuron was in G2 (44/44). Because no G1 cells were labeled by retrobeads and a large fraction of these neurons were labeled by IN markers, it is likely that all G1 neurons are INs. Further, all bead-labeled PNs had G2 properties and were almost never labeled by IN markers. We therefore conclude that G1 neurons are very likely to be INs and G2 neurons are very likely to be PNs, so we will refer to G1 cells as INs and G2 cells as PNs.

In summary, significant differences in most intrinsic electrical properties allow reliable identification of claustral INs and PNs. IN marker gene expression and the presence of long-range anatomical connections in retrobead-labeled neurons support this conclusion.

### Subtypes of claustral PNs

**Figure 6.**
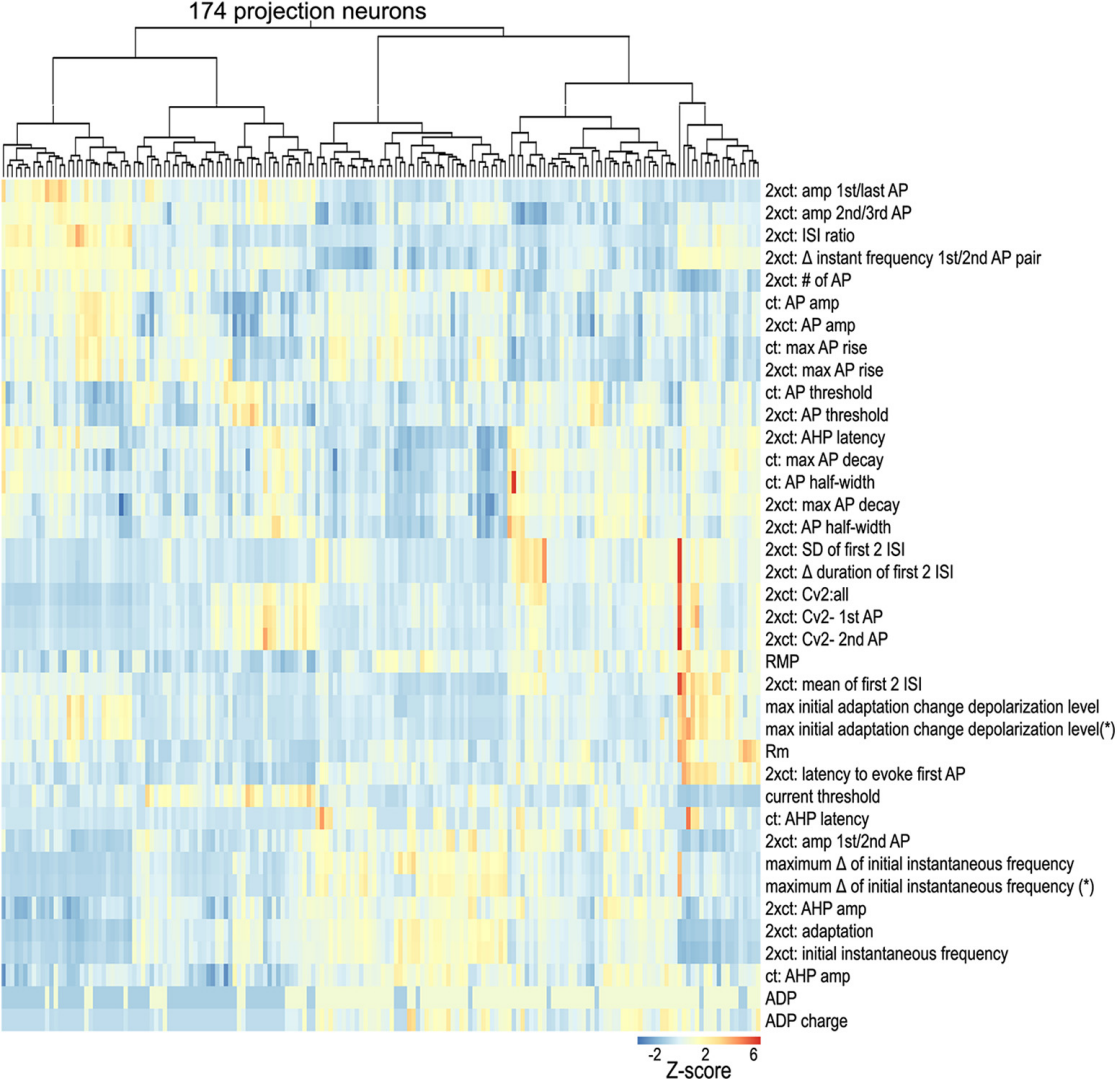
Unsupervised clustering of claustrum PNs. Intrinsic electrical properties of 174 PNs were clustered in an unsupervised manner. Top, Dendrogram showing clusters of PNs. Bottom, Z-scores of extracted intrinsic properties (rows) are shown for each individual cell (column). Hot colors indicate high Z-scores, while cool colors indicate low Z-scores.

**Figure 7.**
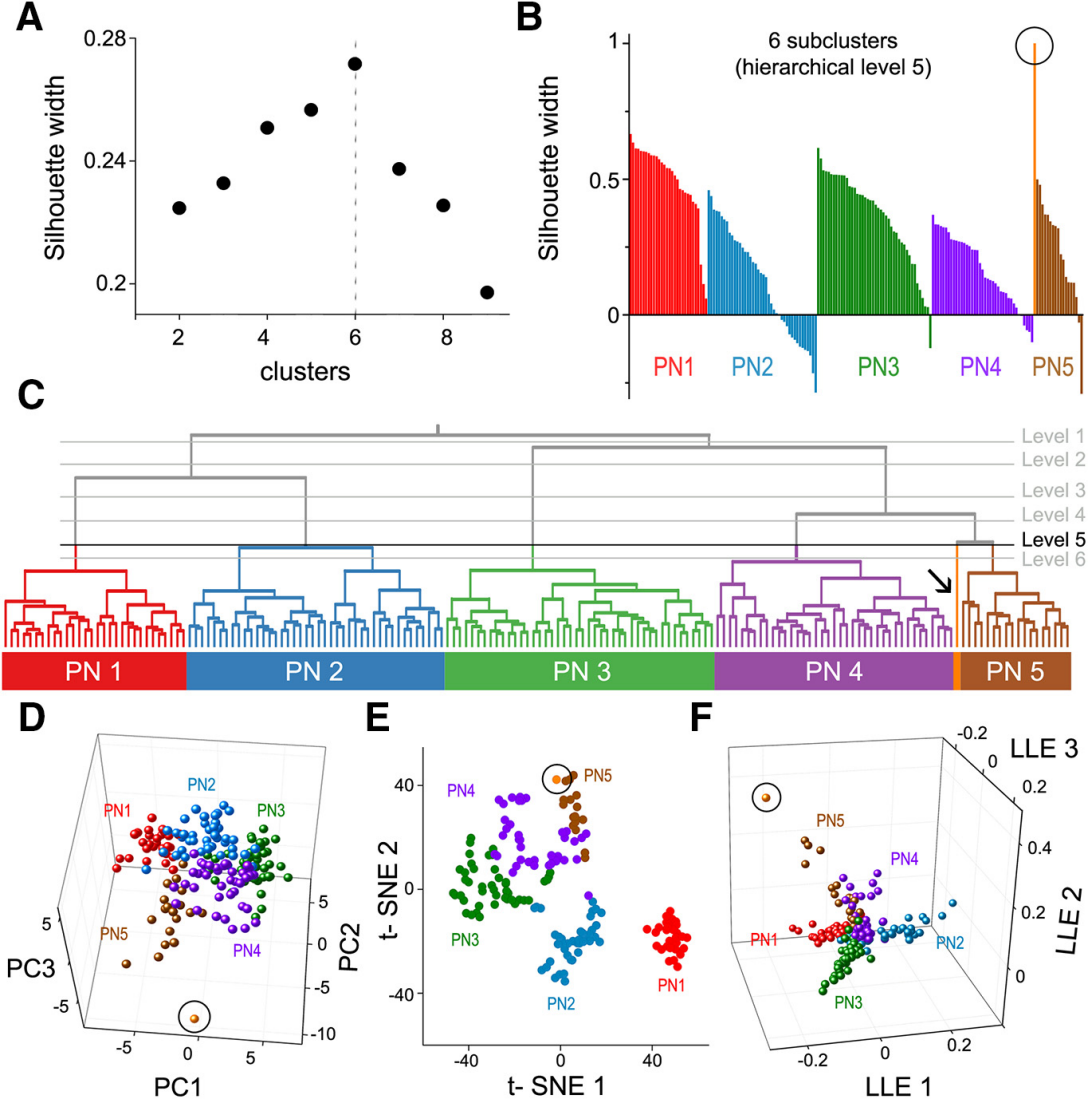
Identification of claustrum PN subtypes. ***A***, Silhouette analyses were performed on data at several hierarchical levels to identify the optimal number of subclusters. The highest silhouette width (SI width) was obtained at hierarchical level 5, containing six clusters. ***B***, SI width of single neurons within each of the six subclusters was ranked; the high SI widths of neurons in subclusters PN1 and PN3 indicates clearest separation from other subclusters. Negative values for SI widths of some PN2 cells suggests the presence of further subclusters. One of the clusters was a single VIP-IN (circled). ***C***, Dendrogram of PN subtypes. ***D–F***, Alternative methods of cell clustering, based on PC (***D***), t-SNE (***E***), and LLE (***F***). All approaches revealed subclusters similar to those generated by hierarchical clustering, confirming the presence of five PN subtypes plus an errant VIP-IN. Same color scheme is used to identify individual neurons in ***B–D***.

To identify subtypes of claustrum PNs and INs, hierarchical clustering was extended to subpopulations of neurons within the IN and PN groups. PNs were clustered using the same intrinsic electrical parameters as above, yielding the dendrogram shown in Figure 6. PNs were separated into two large subclusters that differed mainly in their probability of evincing an ADP at the current threshold, *ct* (first branch: 22%; second branch: 90%), the ratio of the amplitudes of first and last AP at ct*2 (first branch median: 1.43, second branch median: 1.15), and the latency to fire the first AP at ct*2 (first branch median: 16.1 ms; second branch median: 23.9 ms). After this initial bifurcation, the dendrogram split into multiple finer clusters. To determine the optimal number of separate clusters, a silhouette analysis was performed for two clusters (hierarchical level 1) to nine clusters (hierarchical level 8). An average SI width below 0.25 indicates random structure, while higher values indicate clustering (Kaufman and Rousseeuw, 1990). Higher SI values also indicate a higher degree of similarity of neurons within a cluster and better separation from other neurons. The mean SI width peaked at 0.27 for six clusters (Fig. 7*A*), indicating that optimal cell clustering occurred when the left branch was separated into two PN subtypes (PN1/PN2), and the right branch was separated into three PN subtypes (Fig. 7*B*,*C*). The sixth cluster was a single outlier neuron, the VIP-expressing IN, adjacent to PN subtype 5 (Fig. 7*B*, circle, Fig. 7*C*, arrow).

To validate the hierarchical clustering results, neurons were also clustered with three other approaches using their Z-scored intrinsic properties (see Materials and Methods): PC clustering, t-SNE, and LLE. Considering the first three principle components (PCs) of all Z-scored intrinsic properties, PNs segregated in line with the hierarchical clustering results (Fig. 7*D*) and the outlier VIP neuron was separated from the remaining PN clusters (circle). Because the three first PCs account for only ∼60% of the observed variance, there was some overlap between neighboring clusters. In both t-SNE (Fig. 7*E*) and LLE (Fig. 7*F*), PN1–3 neuron subtypes were clearly separated from other groups, while PN4 and PN5 subtypes partially overlapped. This recapitulated the hierarchical clustering, which could only distinguish these subtypes at hierarchical level 4 (Fig. 7*C*). The single VIP-IN (circle) could not be resolved from PN5 neurons using t-SNE but was distinguished in LLE. Taken together, all clustering approaches resulted in a comparable separation of PN subtypes, with neurons being clustered into identical subtypes in all cases.

**Figure 8.**
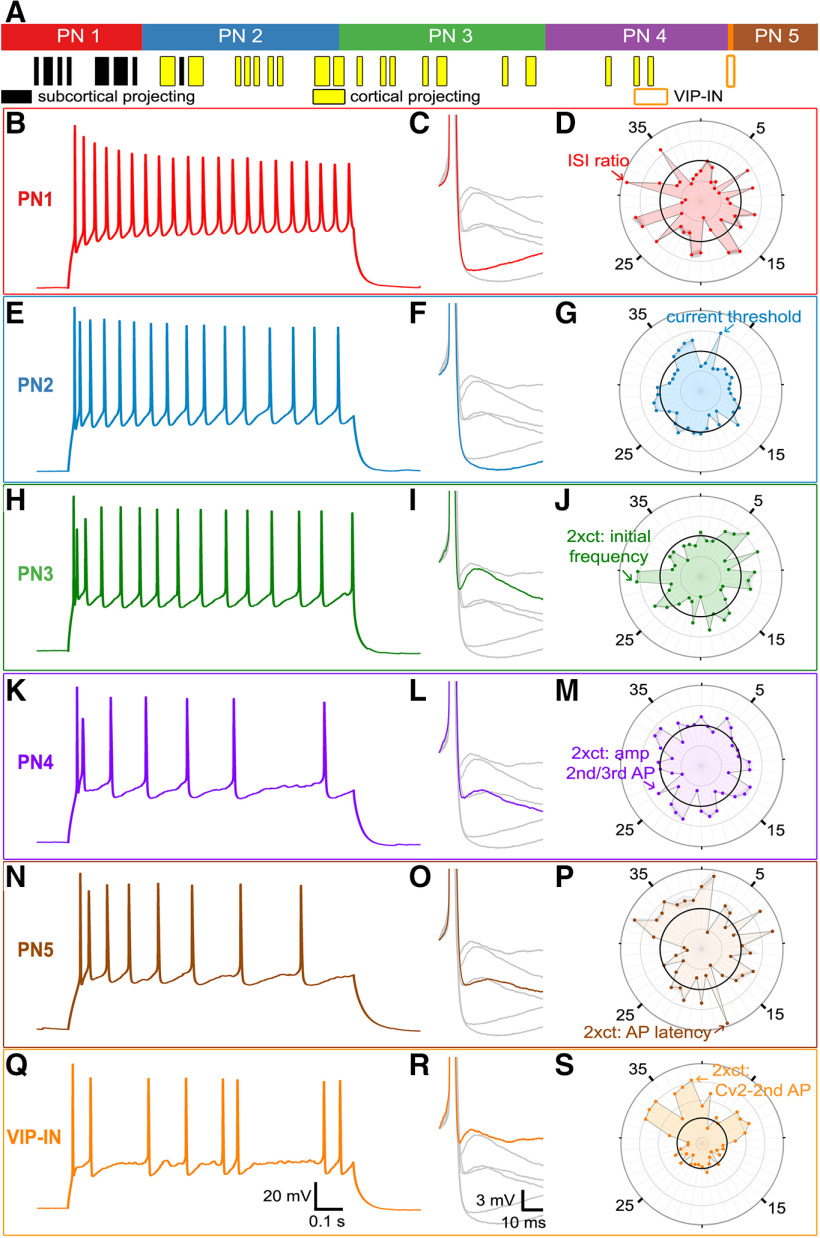
Properties of the five claustrum PN subtypes. ***A***, PNs that were labeled with retrobeads injected into either the cortex (yellow) or subcortex (black) are annotated below the PN subtypes. Neurons that project to the subcortex were highly enriched in the PN1 subcluster, while all claustro-cortical PNs were in PN2–PN4 subclusters. PN5 neurons were not retrogradely labeled, nor was the single VIP-IN misclassified as a PN. ***B–S***, APs and other intrinsic electrical properties of neurons from the six PN subclusters. Left, Trains of APs evoked in the indicated six different types of neurons by depolarizing currents twice as high as the current threshold. Center, Higher-gain traces of individual APs evoked at the current threshold; responses of other PN types are indicated in gray. Right, Radar plot profiles of Z-score means for all 38 intrinsic electrical properties of each PN subtype. Black rings indicate population means. Properties that are most distinct for each PN subtype are indicated by arrows. Properties are numbered as in Tables 3-5.

A comparison of the properties of these PN neuron subtypes is shown in Figure 8. All subtypes, except for PN5 and the outlier VIP-IN, included neurons labeled with retrobeads, indicating that these subtypes are PNs. Remarkably, 92.3% of all subcortical-projecting neurons (12/13) were clustered into PN1, while 100% of the cortical-projecting neurons (28/28) were either PN2, PN3, or PN4. This indicates that the intrinsic electrical properties of claustral PNs allows these cells to be classified into at least two network-specific PN types. PN5 was unlabeled by either retrobeads or IN markers (Fig. 8*A*). Because >98% of the confirmed INs were correctly separated from PNs (Fig. 5*J*), and the only confirmed VIP-IN was flagged as an outlier, we presume that PN5 are PNs that project to targets that were not injected with retrobeads. The single outlier VIP-IN, the only confirmed IN within the PN group, was assigned to group 2 because its AP waveform was similar to that of genuine PNs, but was assigned its own PN subtype because of its unusually irregular AP firing.

A detailed comparison of the properties of all PN subtypes is presented in Tables 3, 4. The medians of the intrinsic electrical properties of all six groups differed significantly from each other (Kruskal–Wallis test), and an overview of their significant differences is shown in Table 5. PN1 neurons had minimal adaptation of AP frequency (Fig. 8*B*) and relatively strong adaptation of AP amplitude over time; they also had no ADP (Fig. 8*C*). A radar plot profile of the mean Z-scores of PN1 intrinsic electrical properties indicated that PN1 differed substantially from the population mean of all PN (Fig. 8*D*, black circle) in many properties, indicating that PN1 neurons were relatively distinct from other PNs. The most distinctive difference between PN1 and other PN subtypes was a relative high ISI ratio (Fig. 8*D*; Table 3, property #31). PN2 neurons rarely generated an ADP when stimulated at the *ct* (Fig. 8*F*), although ADPs were evoked at higher levels of depolarization. This ADP caused modest initial doublet-spiking at higher depolarization levels (Fig. 8*E*) and reduced the amplitude of the second AP in a train. The most distinctive feature of PN2 cells was their high current threshold (Fig. 8*G*; Table 3, property #3), presumably caused by their low membrane resistance (see Table 3). PN3–PN5, as well as the errant VIP-IN, were characterized by a higher probability of generating an ADP at the current threshold (Fig. 8*I*,*L*,*O*,*R*; Tables 3, 4). These cells had variable degrees of initial doublet spiking and a biphasic adaptation of AP amplitude (Fig. 8*H*,*K*,*N*,*Q*). PN3 neurons had the highest initial frequency for their first pair of APs (Fig. 8*J*; Table 3, property #29), indicating pronounced AP doublet firing, and their initial AP burst was followed by relatively constant-frequency AP firing. PN4 and PN5 neurons showed an AP doublet at the onset of the AP train but subsequent AP firing was less consistent than the other PN groups (Fig. 8*K*,*N*). PN4 neurons were readily distinguished by the low amplitude of their second AP relative to the third AP (Fig. 8*K*,*M*; Table 4, property #27). PN5 were distinguished by their delayed AP firing at ct*2 (Fig. 8*P*; Table 4, property #18) and also had relatively high membrane resistance and low current threshold (properties #2 and #3 in Table 4). The VIP-IN also had an ADP at the *ct* and doublet-spiking behavior at higher depolarization levels. However, this cell was distinguished by its high degree of AP firing variability; the Cv2 and the SD of the first two ISIs were larger than for any of the genuine PN (Fig. 8*S*; Table 4, properties #33 and #36).

**Table 3:** Raw data means and median for PN subtypes 1–3

Group		PN1				PN2				PN3			
#	Property	Mean	SEM	Median	MAD	Mean	SEM	Median	MAD	Mean	SEM	Median	MAD
1	RMP (mV)	–73.5	0.51	–73.9	2.01	–74.5	0.34	–74.8	1.59	–71.2	0.40	–71.5	1.92
2	Rm (MΩ)	247	15.6	248	55.4	173	11.4	151	38.4	222	10.7	219	43.0
3	ct	117	8.23	100	40.0	216	13.1	220	60.0	125	8.43	120	40.0
4	ADP probability	0.17	0.07	0.00	0.00	0.26	0.07	0.00	0.00	0.89	0.05	1.00	0.00
5	Mean ADP value (mV)	0.08	0.05	0.00	0.00	0.24	0.08	0.00	0.00	1.54	0.15	1.46	0.70
6	Max initial adaptation change(Hz/pA)	0.47	0.02	0.44	0.08	1.25	0.10	0.99	0.35	2.99	0.13	3.18	0.48
7	Max adaptation change relative to ct	2.01	0.10	1.95	0.45	1.35	0.04	1.28	0.09	1.30	0.03	1.25	0.10
8	Initial adaptation change (Hz/pA) (2)	0.47	0.02	0.44	0.08	1.16	0.09	0.91	0.27	2.58	0.14	2.74	0.67
9	Max adaptation change relativeto ct (2)	2.01	0.10	1.95	0.45	1.37	0.04	1.29	0.08	1.34	0.03	1.33	0.13
10	AHP amplitude (mV)	–14.9	0.50	–14.8	2.17	–14.6	0.51	–14.5	1.99	–10.1	0.38	–10.2	1.59
11	AHP latency (ms)	12.3	1.51	9.22	2.77	13.1	1.99	6.57	2.27	64.4	10.3	56.5	51.8
12	Max AP rise (mV/ms)	225	6.77	222	25.0	183	5.35	191	30.5	216	5.80	215	22.7
13	Max AP decay (mV/ms)	–39.7	1.23	–39.1	4.69	–38.2	0.93	–38.3	3.91	–44.2	0.80	–43.8	3.13
14	AP half-width (ms)	1.95	0.05	1.94	0.16	1.81	0.04	1.76	0.14	1.57	0.03	1.57	0.13
15	AP threshold (mV)	–35.6	0.53	–36.1	1.98	–32.4	0.47	–32.1	1.95	–34.8	0.27	–34.8	1.11
16	AP amplitude (mV)	81.4	0.76	81.6	2.82	71.8	0.73	71.5	3.08	77.1	0.46	77.2	1.68
17	2xct: number of AP	20.1	0.63	20.0	2.00	15.0	0.69	14.5	2.50	17.8	0.64	17.5	2.50
18	2xct: latency to first AP	17.5	0.72	17.8	3.02	15.3	0.70	15.2	2.58	21.5	0.82	21.3	3.44
19	2xct: AHP amplitude (mV)	–11.1	0.56	–11.3	2.59	–6.48	0.56	–6.15	2.06	–2.98	0.40	–2.84	1.40
20	2xct: AHP amplitude latency (ms)	4.41	0.12	4.34	0.32	4.12	0.08	4.06	0.32	3.37	0.06	3.39	0.28
21	2xct: max AP rise (mV/ms)	227	7.41	224	24.2	200	7.32	190	35.9	210	6.29	205	30.5
22	2xct: max AP decay (mV/ms)	–45.7	1.43	–44.5	5.47	–43.6	1.07	–43.0	4.69	–48.5	0.99	–48.4	4.69
23	2xct: AP half-width (ms)	1.63	0.03	1.64	0.09	1.78	0.04	1.77	0.17	1.48	0.03	1.46	0.11
24	2xct: AP threshold (mV)	–37.3	0.56	–37.7	2.47	–35.7	0.74	–36.7	2.96	–36.8	0.26	–36.7	1.40
25	2xct: AP amplitude (mV)	78.8	0.80	78.7	2.54	72.6	0.75	73.9	2.64	75.5	0.50	75.9	2.16
26	2xct: 1st/2nd AP ratio	1.17	0.02	1.14	0.03	1.30	0.02	1.30	0.09	1.44	0.02	1.41	0.10
27	2xct: 2nd/3rd AP ratio	1.05	0.01	1.05	0.02	0.99	0.01	0.99	0.04	0.92	0.01	0.93	0.07
28	2xct: 1st /last AP ratio	1.60	0.05	1.56	0.20	1.38	0.03	1.32	0.12	1.18	0.01	1.16	0.07
29	2xct: initial instant frequency	35.5	1.15	34.7	3.25	71.4	2.72	72.7	11.2	101	2.87	100	13.0
30	2xct: max adaptation (Hz)	17.5	1.20	18.2	3.50	58.7	2.66	59.1	13.2	87.0	2.81	86.0	11.9
31	2xct: ISI ratio	0.52	0.03	0.50	0.07	0.17	0.01	0.17	0.06	0.15	0.01	0.14	0.03
32	2xct: average initial burst interva (ms) first 3 AP	33.8	1.00	33.8	3.89	21.2	1.08	19.7	3.59	18.9	1.28	16.0	4.54
33	2xct: SD of average initial burstinterval first 3 AP	6.64	0.69	5.88	1.87	8.65	0.85	7.73	2.61	12.3	1.56	7.95	4.07
34	2xct: change of initial interspikeinterval (ms)	9.29	1.00	8.32	2.64	12.2	1.20	10.9	3.69	17.4	2.21	11.2	5.76
35	2xct: initial change of instantfrequency	–8.65	0.85	–8.74	2.24	–30.5	1.73	–30.6	5.09	–54.3	2.27	–56.0	12.4
36	2xct: Cv2:all	0.08	0.01	0.07	0.01	0.24	0.02	0.21	0.09	0.17	0.01	0.16	0.04
37	2xct: Cv2-1st AP	0.06	0.00	0.06	0.01	0.22	0.02	0.18	0.10	0.12	0.01	0.11	0.03
38	2xct: Cv2-1st/2nd AP	0.06	0.00	0.05	0.01	0.21	0.02	0.15	0.09	0.09	0.01	0.08	0.02

Means are indicated with their SEM, while medians are indicated with their median absolute deviation (MAD).

**Table 4:** Raw data means and medians for PN subytpes 4 and 5, as well as the outlier VIP-IN

Group		PN4				PN5				Outlier VIP-IN
#	Property	Mean	SEM	Median	MAD	Mean	SEM	Median	MAD	value
1	RMP (mV)	–70.4	0.39	–70.5	1.44	–68.1	0.88	–68.7	1.44	–64.9
2	Rm (MΩ)	244	13.2	238	59.6	448	36.5	399	82.7	755
3	ct	118	7.84	100	20.0	49.4	3.83	40.0	15.0	30.0
4	ADP probability	0.95	0.04	1.00	0.00	0.83	0.09	1.00	0.00	1.00
5	Mean ADP value (mV)	1.26	0.16	1.12	0.72	1.14	0.23	1.42	0.89	0.99
6	Max initial adaptation change (Hz/pA)	1.70	0.11	1.47	0.36	1.20	0.11	1.05	0.26	5.67
7	Max adaptation change relative to ct	1.51	0.04	1.50	0.17	2.36	0.17	2.29	0.29	3.33
8	Initial adaptation change (Hz/pA) (2)	1.45	0.10	1.42	0.40	1.20	0.11	1.05	0.26	5.67
9	Max adaptation change relative to ct (2)	1.59	0.05	1.50	0.17	2.53	0.17	2.33	0.33	3.33
10	AHP amplitude (mV)	–11.1	0.54	–10.8	2.12	–12.5	0.73	–11.7	2.15	–7.78
11	AHP latency (ms)	66.4	8.42	63.6	36.7	66.5	22.4	8.75	4.33	4.12
12	Max AP rise (mV/ms)	170	4.43	167	14.1	173	7.56	164	15.2	172
13	Max AP decay (mV/ms)	–34.5	0.82	–34.4	3.13	–33.6	1.07	–33.6	3.91	–48.4
14	AP half-width (ms)	2.02	0.06	1.93	0.17	1.97	0.06	2.00	0.23	1.61
15	AP threshold (mV)	–33.1	0.47	–33.1	2.18	–33.0	0.49	–33.1	1.69	–39.8
16	AP amplitude (mV)	74.1	0.64	73.9	3.02	73.8	0.67	74.2	3.09	76.7
17	2xct: number of AP	12.8	0.51	13.0	3.00	10.1	0.91	9.50	2.50	8.00
18	2xct: latency to first AP	25.1	0.95	24.9	3.26	38.7	2.46	42.0	5.71	17.0
19	2xct: AHP amplitude (mV)	–3.60	0.61	–3.57	1.99	–8.92	0.62	–8.56	1.74	–2.98
20	2xct: AHP amplitude latency (ms)	4.20	0.08	4.14	0.36	4.48	0.11	4.53	0.25	3.86
21	2xct: max AP rise (mV/ms)	161	4.24	166	18.8	170	7.11	166	16.4	169
22	2xct: max AP decay (mV/ms)	–37.0	0.80	–37.5	3.13	–37.9	1.16	–37.5	1.56	–46.9
23	2xct: AP half-width (ms)	1.86	0.05	1.77	0.16	1.74	0.05	1.75	0.14	1.64
24	2xct: AP threshold (mV)	–34.5	0.52	–34.7	2.24	–34.5	0.46	–34.5	0.99	–41.7
25	2xct: AP amplitude (mV)	72.2	0.59	72.2	2.65	73.0	0.67	72.7	2.01	77.3
26	2xct: 1st/2nd AP ratio	1.42	0.03	1.40	0.11	1.16	0.02	1.18	0.05	1.15
27	2xct: 2nd/3rd AP ratio	0.86	0.01	0.87	0.05	0.95	0.01	0.95	0.02	1.01
28	2xct: 1st /last AP ratio	1.18	0.01	1.16	0.05	1.13	0.01	1.11	0.04	1.18
29	2xct: initial instant frequency	68.2	2.40	70.2	8.25	29.1	2.80	29.0	10.19	16.0
30	2xct: max adaptation (Hz)	58.0	2.43	58.3	9.69	20.5	2.30	18.6	8.62	2.49
31	2xct: ISI ratio	0.15	0.01	0.15	0.04	0.30	0.02	0.31	0.05	0.46
32	2xct: average initial burst interval (ms) first 3 AP	33.3	1.98	29.3	6.58	57.8	5.14	50.7	14.6	133
33	2xct: SD of average initial burst interval first 3 AP	25.1	2.55	21.5	7.38	23.8	1.89	22.6	5.20	99.4
34	2xct: change of initial interspike interval (ms)	35.5	3.61	30.5	10.4	33.6	2.68	31.9	7.36	141
35	2xct: initial change of instant frequency	–44.7	2.33	–43.0	10.5	–14.0	1.62	–13.4	6.19	–11.1
36	2xct: Cv2:all	0.26	0.02	0.23	0.07	0.27	0.03	0.27	0.09	0.86
37	2xct: Cv2-1st AP	0.17	0.01	0.16	0.04	0.22	0.04	0.21	0.09	0.82
38	2xct: Cv2-1st/2nd AP	0.15	0.01	0.13	0.05	0.20	0.03	0.18	0.08	0.92

Means are indicated with their SEM, while medians are indicated with their median absolute deviation (MAD).

**Table 5:** Statistical comparison of all PN subtypes (including the outlier VIP-IN)

Group		Kruskal–Wallis			Dunn’s multiple comparison test														
#	Property	*p* value	*H* value	η^2^	PN1vsPN2	PN1vsPN3	PN1vsPN4	PN1vsPN5	PN1vsVIP	PN2vsPN3	PN2vsPN4	PN2vsPN5	PN2vsVIP	PN3vsPN4	PN3vsPN5	PN3vsVIP	PN4vsPN5	PN4vsVIP	PN5vsVIP
1	RMP (mV)	3.0E-13	67.8	0.37	1.0E+00	3.1E-02	7.5E-04	7.6E-06	6.2E-01	1.6E-05	6.2E-08	1.6E-09	2.7E-01	1.0E+00	9.1E-02	1.0E+00	1.0E+00	1.0E+00	1.0E+00
2	Rm (MΩ)	4.6E-11	57.2	0.31	4.2E-03	1.0E+00	1.0E+00	1.5E-03	1.0E+00	5.1E-02	2.1E-03	8.9E-12	2.4E-01	1.0E+00	8.9E-06	1.0E+00	5.0E-04	1.0E+00	1.0E+00
3	ct	2.1E-15	78.0	0.43	1.3E-04	1.0E+00	1.0E+00	1.7E-04	1.0E+00	8.4E-05	2.1E-05	0.0E+00	1.4E-01	1.0E+00	9.9E-06	1.0E+00	8.0E-05	1.0E+00	1.0E+00
4	ADP probability	1.1E-16	83.8	0.47	1.0E+00	6.3E-09	5.5E-10	6.5E-05	1.0E+00	4.1E-08	3.3E-09	4.6E-04	1.0E+00	1.0E+00	1.0E+00	1.0E+00	1.0E+00	1.0E+00	1.0E+00
5	ADP mean	3.3E-16	82.1	0.46	1.0E+00	1.9E-10	3.3E-08	1.8E-04	1.0E+00	9.0E-10	2.1E-07	1.2E-03	1.0E+00	1.0E+00	1.0E+00	1.0E+00	1.0E+00	1.0E+00	1.0E+00
6	Max initial adaptationchange (Hz/pA)	0.0E+00	121	0.69	6.0E-05	0.0E+00	6.6E-10	2.4E-03	3.4E-02	1.5E-09	3.8E-01	1.0E+00	7.1E-01	6.7E-04	1.4E-05	1.0E+00	1.0E+00	1.0E+00	8.0E-01
7	Max adaptationchange relativeto ct	1.9E-15	78.3	0.44	8.6E-08	1.5E-09	8.9E-03	1.0E+00	1.0E+00	1.0E+00	1.8E-01	1.1E-07	4.7E-01	2.3E-02	3.9E-09	3.3E-01	2.6E-03	1.0E+00	1.0E+00
8	Initial adaptationchange (Hz/pA) (2)	0.0E+00	106	0.6	2.3E-05	0.0E+00	2.9E-09	2.0E-04	3.5E-02	2.9E-07	1.0E+00	1.0E+00	8.3E-01	3.2E-03	2.2E-03	1.0E+00	1.0E+00	1.0E+00	1.0E+00
9	Max adaptationchange relativeto ct (2)	0.0E+00	88.7	0.5	2.5E-08	3.9E-09	8.3E-02	1.0E+00	1.0E+00	1.0E+00	8.5E-03	3.5E-10	3.8E-01	2.8E-03	6.3E-11	3.3E-01	1.3E-03	1.0E+00	1.0E+00
10	AHP amplitude (mV)	8.3E-12	60.8	0.33	1.0E+00	2.8E-08	9.2E-05	2.4E-01	5.1E-01	1.5E-08	1.2E-04	4.4E-01	6.4E-01	1.0E+00	1.8E-01	1.0E+00	1.0E+00	1.0E+00	1.0E+00
11	AHP latency (ms)	3.7E-04	22.8	0.11	1.0E+00	1.0E+00	5.4E-02	1.0E+00	1.0E+00	1.5E-01	2.2E-04	7.4E-01	1.0E+00	9.7E-01	1.0E+00	1.0E+00	1.0E+00	7.4E-01	1.0E+00
12	Max AP rise (mV/ms)	2.8E-10	53.4	0.29	5.3E-04	1.0E+00	2.7E-07	1.6E-04	1.0E+00	3.9E-03	1.0E+00	1.0E+00	1.0E+00	1.7E-06	1.1E-03	1.0E+00	1.0E+00	1.0E+00	1.0E+00
13	Max AP decay (mV/ms)	3.1E-12	62.9	0.34	1.0E+00	5.2E-02	1.2E-02	2.1E-02	1.0E+00	6.6E-04	7.4E-02	1.0E-01	1.0E+00	1.1E-10	6.6E-08	1.0E+00	1.0E+00	5.1E-01	4.0E-01
14	AP half-width (ms)	8.4E-13	65.6	0.36	3.0E-01	4.1E-08	1.0E+00	1.0E+00	1.0E+00	1.2E-03	3.4E-02	3.7E-01	1.0E+00	5.1E-11	1.7E-06	1.0E+00	1.0E+00	1.0E+00	1.0E+00
15	AP threshold (mV)	1.9E-06	34.5	0.18	7.2E-05	1.0E+00	3.5E-03	1.4E-02	1.0E+00	2.1E-03	1.0E+00	1.0E+00	4.9E-01	7.0E-02	1.6E-01	1.0E+00	1.0E+00	7.9E-01	6.8E-01
16	AP amplitude (mV)	1.1E-13	69.8	0.39	6.6E-13	2.2E-02	1.2E-07	2.9E-06	1.0E+00	1.6E-05	1.0E+00	1.0E+00	1.0E+00	4.7E-02	6.4E-02	1.0E+00	1.0E+00	1.0E+00	1.0E+00
17	2xct: number of AP	1.1E-13	66.9	0.37	1.6E-04	4.8E-01	7.1E-09	4.1E-10	2.6E-01	1.7E-01	5.9E-01	1.4E-02	1.0E+00	7.5E-05	1.9E-06	8.9E-01	1.0E+00	1.0E+00	1.0E+00
18	2xct: latencyto first AP	0.0E+00	87.7	0.49	1.0E+00	1.6E-01	6.1E-05	5.5E-09	1.0E+00	1.3E-04	5.2E-10	4.7E-14	1.0E+00	2.9E-01	9.6E-05	1.0E+00	1.3E-01	1.0E+00	6.7E-01
19	2xct: AHP amplitude (mV)	1.1E-16	85.0	0.48	4.3E-04	4.0E-14	2.6E-10	1.0E+00	8.2E-01	8.2E-04	6.6E-02	2.9E-01	1.0E+00	1.0E+00	6.7E-07	1.0E+00	8.4E-05	1.0E+00	1.0E+00
20	2xct: AHP amplitudelatency (ms)	1.1E-13	69.8	0.39	9.5E-01	3.1E-10	1.0E+00	1.0E+00	1.0E+00	1.8E-06	1.0E+00	3.2E-01	1.0E+00	5.5E-08	2.4E-09	1.0E+00	1.0E+00	1.0E+00	1.0E+00
21	2xct: max AP rise(mV/ms)	5.3E-10	52.0	0.28	1.4E-01	1.0E+00	1.0E-08	1.7E-04	1.0E+00	1.0E+00	1.2E-03	2.1E-01	1.0E+00	3.3E-06	1.0E-02	1.0E+00	1.0E+00	1.0E+00	1.0E+00
22	2xct: max APdecay (mV/ms)	3.3E-12	62.8	0.34	1.0E+00	1.0E+00	4.9E-05	5.2E-03	1.0E+00	4.7E-02	7.8E-04	4.5E-02	1.0E+00	4.2E-11	2.1E-06	1.0E+00	1.0E+00	1.0E+00	1.0E+00
23	2xct: AP half-width (ms)	8.9E-11	55.8	0.3	2.4E-01	6.6E-02	1.6E-02	1.0E+00	1.0E+00	1.0E-07	1.0E+00	1.0E+00	1.0E+00	3.5E-10	8.1E-04	1.0E+00	1.0E+00	1.0E+00	1.0E+00
24	2xct: AP threshold (mV)	8.0E-05	26.3	0.13	1.0E+00	1.0E+00	5.7E-03	9.0E-03	1.0E+00	1.0E+00	2.6E-01	2.2E-01	1.0E+00	1.3E-02	2.1E-02	1.0E+00	1.0E+00	5.5E-01	4.0E-01
25	2xct: AP amplitude (mV)	8.3E-09	46.2	0.25	7.8E-06	1.9E-01	9.5E-08	1.8E-04	1.0E+00	6.9E-02	1.0E+00	1.0E+00	1.0E+00	2.8E-03	1.5E-01	1.0E+00	1.0E+00	1.0E+00	1.0E+00
26	2xct: 1st/2nd AP ratio	4.4E-16	81.4	0.46	3.6E-03	1.6E-11	6.2E-09	1.0E+00	1.0E+00	2.7E-03	6.0E-02	2.5E-02	1.0E+00	1.0E+00	2.1E-08	9.2E-01	1.3E-06	1.0E+00	1.0E+00
27	2xct: 2nd/3rdAP ratio	0.0E+00	95.3	0.54	6.9E-03	8.6E-10	0.0E+00	7.7E-05	1.0E+00	1.4E-02	7.8E-09	9.6E-01	1.0E+00	3.6E-02	1.0E+00	1.0E+00	3.8E-02	1.0E+00	1.0E+00
28	2xct: 1st /lastAP ratio	0.0E+00	90.2	0.51	5.4E-01	6.7E-11	2.4E-10	2.5E-10	1.0E+00	2.0E-06	5.0E-06	1.4E-06	1.0E+00	1.0E+00	1.0E+00	1.0E+00	1.0E+00	1.0E+00	1.0E+00
29	2xct: initial instantfrequency	0.0E+00	128	0.73	7.6E-07	0.0E+00	1.8E-05	1.0E+00	1.0E+00	1.4E-04	1.0E+00	3.0E-06	1.0E+00	1.4E-05	0.0E+00	8.3E-02	3.8E-05	1.0E+00	1.0E+00
30	2xct: maxadaptation (Hz)	0.0E+00	127	0.73	6.9E-08	0.0E+00	2.1E-07	1.0E+00	1.0E+00	2.7E-04	1.0E+00	4.3E-05	9.5E-01	2.4E-04	1.3E-14	8.4E-02	8.1E-05	1.0E+00	1.0E+00
31	2xct: ISI ratio	0.0E+00	97.1	0.55	2.0E-11	0.0E+00	4.3E-13	6.1E-01	1.0E+00	1.0E+00	1.0E+00	1.7E-03	1.0E+00	1.0E+00	1.9E-05	1.0E+00	2.1E-04	1.0E+00	1.0E+00
32	2xct: average initial burstinterval (ms) first 3 AP	0.0E+00	92.1	0.52	1.2E-05	2.8E-08	1.0E+00	3.4E-01	1.0E+00	1.0E+00	3.5E-04	6.0E-10	3.7E-01	1.1E-06	8.7E-13	1.9E-01	1.9E-02	1.0E+00	1.0E+00
33	2xct: SD of averageinitial burst intervalfirst 3 AP	1.9E-14	73.5	0.41	1.0E+00	2.6E-01	1.2E-09	1.1E-07	2.2E-01	1.0E+00	1.6E-07	7.6E-06	4.8E-01	5.9E-05	5.2E-04	8.7E-01	1.0E+00	1.0E+00	1.0E+00
34	2xct: change of initialinterspike interval (ms)	1.9E-14	73.5	0.41	1.0E+00	2.6E-01	1.2E-09	1.1E-07	2.2E-01	1.0E+00	1.6E-07	7.6E-06	4.8E-01	5.9E-05	5.2E-04	8.7E-01	1.0E+00	1.0E+00	1.0E+00
35	2xct: initial changeof instant frequency	0.0E+00	123	0.7	1.7E-05	0.0E+00	3.2E-13	1.0E+00	1.0E+00	8.6E-06	2.7E-02	2.5E-02	1.0E+00	1.0E+00	3.4E-11	6.7E-01	4.6E-07	1.0E+00	1.0E+00
36	2xct: Cv2:all	5.6E-16	80.8	0.45	1.8E-11	5.8E-06	8.0E-14	3.3E-09	3.8E-02	3.2E-01	1.0E+00	1.0E+00	1.0E+00	2.3E-02	2.0E-01	9.7E-01	1.0E+00	1.0E+00	1.0E+00
37	2xct: Cv2-1st AP	9.1E-15	75.1	0.42	9.2E-13	3.0E-05	1.3E-10	1.7E-08	4.6E-02	2.9E-02	1.0E+00	1.0E+00	1.0E+00	2.3E-01	2.0E-01	9.3E-01	1.0E+00	1.0E+00	1.0E+00
38	2xct: Cv2-1st/2nd AP	1.3E-13	69.6	0.38	5.1E-10	1.3E-01	5.4E-08	1.7E-07	9.7E-02	1.1E-04	1.0E+00	1.0E+00	1.0E+00	3.2E-03	1.6E-03	5.0E-01	1.0E+00	1.0E+00	1.0E+00

In summary, our analysis of intrinsic electrical properties revealed multiple subtypes of claustral PNs that differed in their projection targets: cortical (PN2, PN3, PN4) or subcortical (PN1) areas. The target of PN5 cells remains to be determined.

### Subtypes of claustral IN

**Figure 9.**
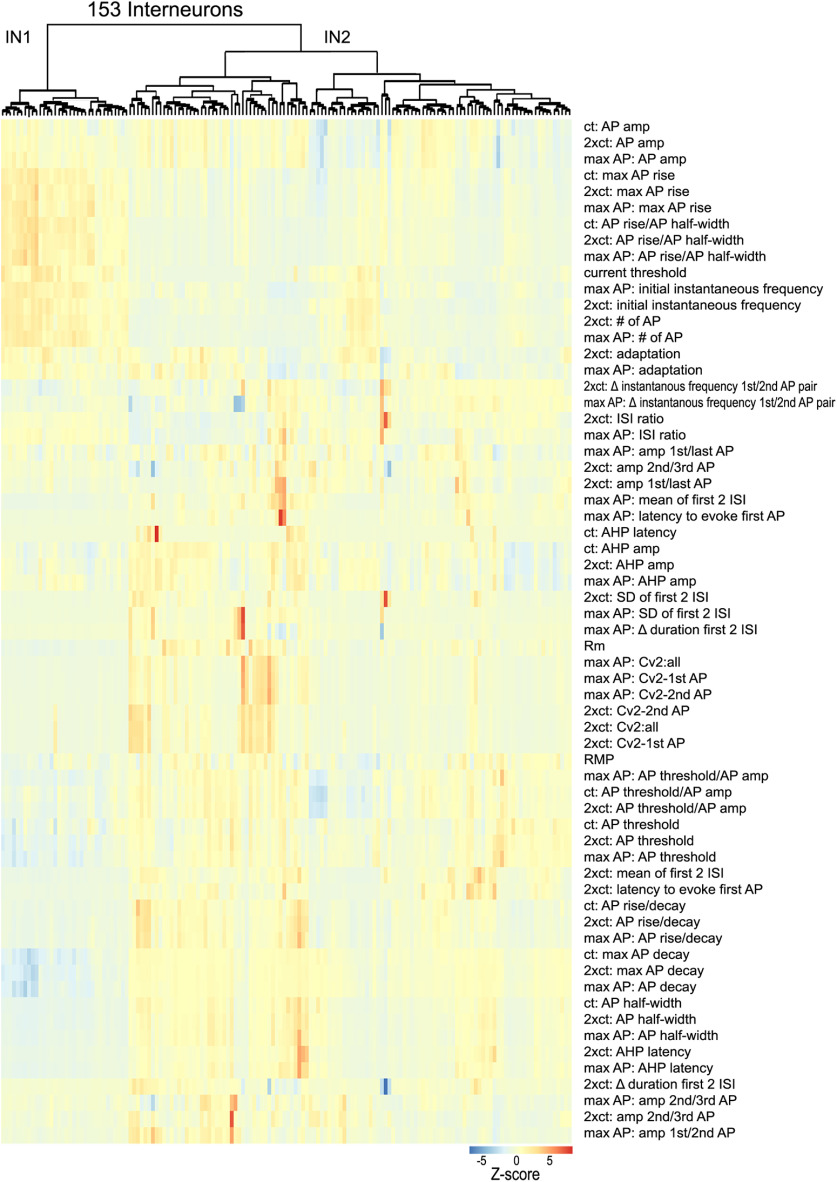
Unsupervised clustering of claustrum IN. A total of 63 intrinsic electrical properties were analyzed for 153 INs at three different depolarization levels: *ct*, 2x*ct*, and level that evoked maximal AP firing and then clustered in an unsupervised manner. Top, Dendrogram showing clusters of IN. Bottom, Z-scores of intrinsic properties (rows) are shown for each individual cell (column). Hot colors indicate high Z-scores, while cool colors indicate low Z-scores.

INs in the CNS are diverse and consist of numerous subtypes (Ascoli et al., 2008). To identify claustrum IN subtypes, we analyzed 63 intrinsic electrical properties and measured responses to three different depolarizing currents: at threshold, twice the threshold current, and at levels that evoked maximal AP firing. Hierarchical clustering of these IN intrinsic properties, as done for PNs, revealed one smaller cluster of INs on the left side of the dendrogram (IN1) that was distinct and differed in most properties from a larger cluster on the right (IN2; Fig. 9). Neurons in the smaller cluster had higher AP rise rates, more negative AP decay rates, and shorter AP half-widths.

**Figure 10.**
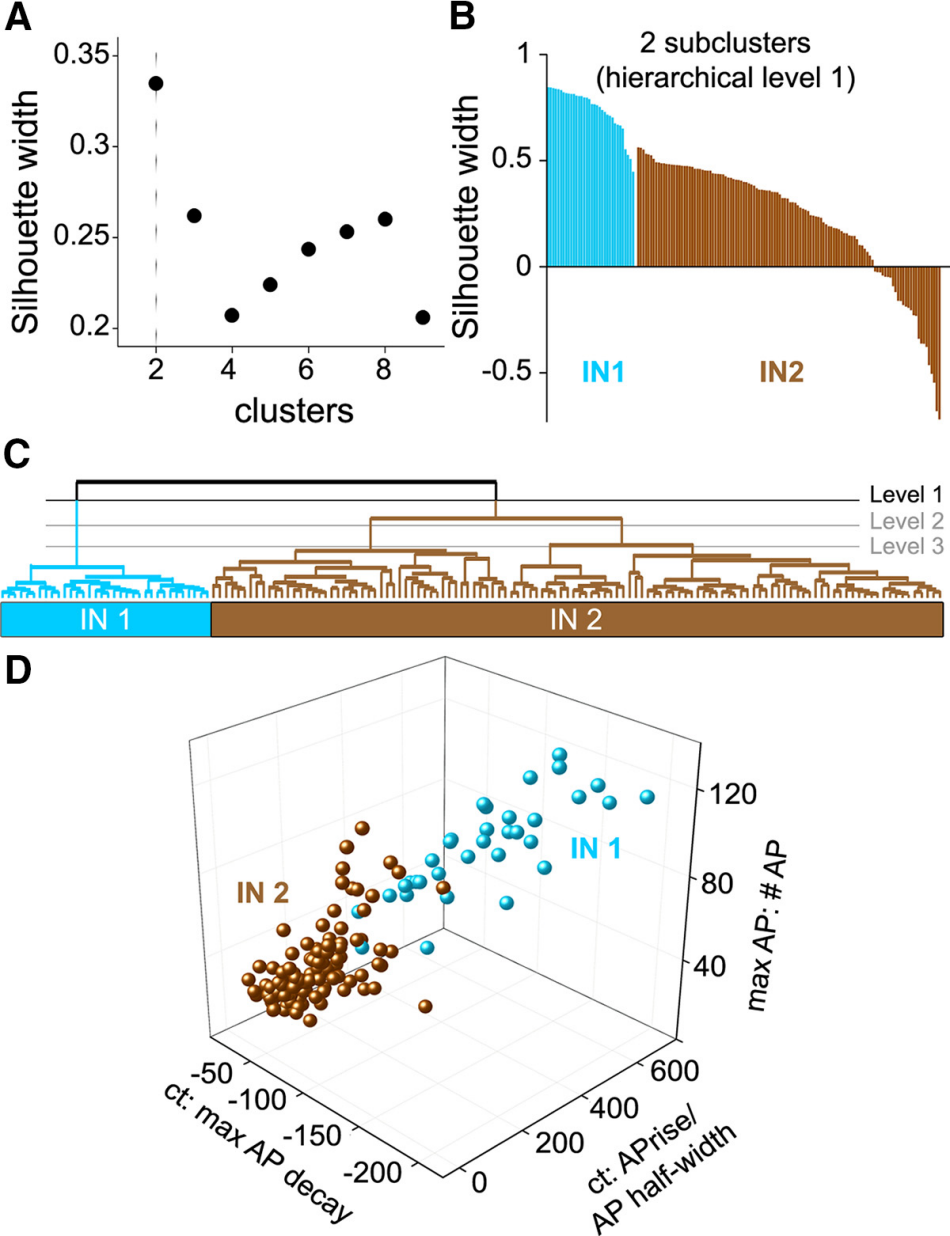
Claustrum IN subtypes. ***A***, Silhouette analyses were performed on data at several hierarchical levels to identify the optimal number of subclusters. The highest silhouette width (SI width) was obtained at hierarchical level 1, containing two clusters. ***B***, SI width of single neurons within each of the two subclusters was ranked; the high SI widths of neurons in subcluster IN1 indicates clear separation from IN2, while the high variability of IN2 neurons indicates a heterogeneous subpopulation. ***C***, Dendrogram of IN subtypes. ***D***, Comparison of individual IN, showing the three features that differed the most between IN1 and IN2.

To determine whether additional subgroups of IN could be distinguished, mean SI width was calculated for two (hierarchical level 1) to nine (hierarchical level 8) IN subclusters (Fig. 10*A*). The highest mean SI width was 0.33, at hierarchical level 1, with an average SI value of 0.26 at hierarchical level 2 and values below the threshold of 0.25 for larger numbers of clusters. The maximum SI value at hierarchical level 1 reflects the two main subtypes readily apparent in Figure 9, while a further subclassification into three groups is plausible. At hierarchical level 1, the first cell cluster accounted for 28.8% of all neurons (34/152), and their single-neuron SI values were generally high, indicating relatively homogenous intrinsic properties that allowed for clear separation of IN1 from IN2 (Fig. 10*B*). Neurons within the second cluster accounted for 71.2% of all IN and their single-neuron SI values varied very broadly, indicating a more heterogenous population. A plot of the three most distinctive properties, determined from their absolute differences within subpopulation Z-score means, again demonstrated that the two IN subtypes were clearly separable (Fig. 10*C*,*D*).

**Figure 11.**
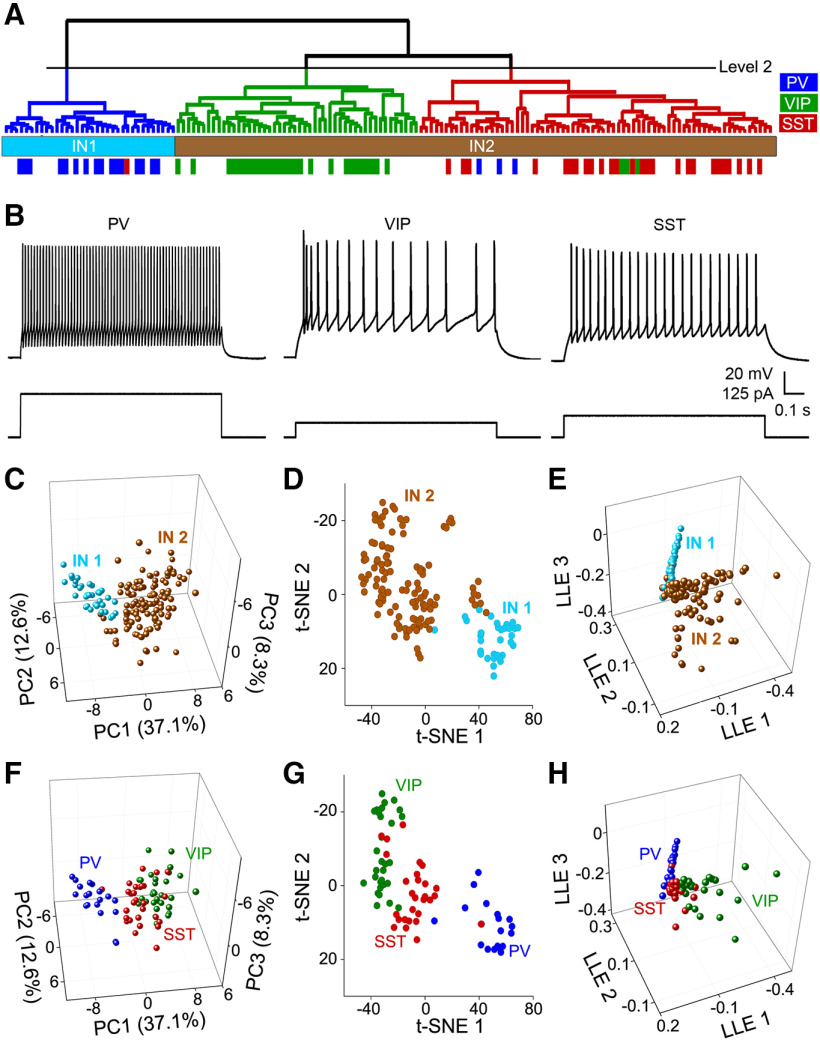
Identification of claustrum IN subtypes. ***A***, Dendrogram from Figure 10*C*, annotated with identified INs that expressed PV (blue), SST (red), or VIP (green). Neurons that expressed PVs were mostly clustered into IN1, identifying IN1 as PV-INs. Aside from a single SST-IN, all confirmed VIP-INs and SST-INs were in IN2, indicating that this subcluster consists of multiple IN subtypes. ***B***, Representative examples of responses of the three different IN subtypes to depolarizing currents twice as high as the current threshold. ***C***, Range of intrinsic electrical properties of claustral IN expressed by the first three PCs (PC1–3). Numbers in parentheses indicate the percentage of variance explained by the PC. ***D***, ***E***, Alternative IN clustering based on t-SNE (***D***) or LLE (***E***). Both alternative methods yielded subclusters that separated IN1 and IN2. ***F–H***, Clustering of the same dataset shown in ***C–E***, indicating neurons with PV (blue), SST (red), or VIP (green) expression. Clustering was done using PC analysis (***F***), t-SNE (***G***), or LLE (***H***), and all three approaches showed that IN subtypes differed in their intrinsic parameters and clustered into separate groups.

Cortical INs consist of at least three distinct types: those expressing PV, SST, or the type 3a serotonin receptor (5HT3aR), with the latter including a large subpopulation of VIP-IN (Lee et al., 2010; Rudy et al., 2011). We next asked whether expression of PV, SST, or VIP correlated with claustrum IN intrinsic electrical properties by mapping IN marker expression onto the hierarchical clustering dendrogram (Fig. 11*A*). Most confirmed PV-INs (16/19, 84.2%) were clustered into IN1, while the remainder (3/19) were in IN2. Conversely, 96.4% (27/28) of all confirmed SST-INs were clustered into IN2, while the single remaining SST-IN had IN1 characteristics. All VIP-INs (30/30) clustered into IN2. These results indicate that the intrinsic properties of PV-INs were sufficiently distinct from those of VIP-INs and SST-INs to separate PV-INs into a distinct cluster. Representative responses of a PV-IN (Fig. 11*B*, left), a VIP-IN (Fig. 11*B*, middle), and a SST-IN (Fig. 11*B*, right) to depolarizing current pulses illustrate that PV-INs were clearly distinguished by their rapid and regular AP firing, as previously described (Kim et al., 2016; White and Mathur, 2018). A detailed analysis of the first three PCs of all Z-scored electrical parameters showed that the PV-enriched IN1 cluster separated fairly well from the IN2 cluster enriched in VIP-INs and SST-INs (Fig. 11*C*). The alternative clustering approaches t-SNE (Fig. 11*D*) and LLE (Fig. 11*E*) yielded identical separation of IN1 and IN2 neurons. While differences in the intrinsic properties of PV-INs and VIP-INs were large enough to prevent overlap in feature space, SST-INs overlapped with both clusters. This heterogeneity and overlap, particularly within IN2, the combined SST-IN and VIP-IN cluster, is responsible for the blurring of VIP-IN and SST-IN subclusters and causes the mean SI width at two clusters to be higher than that for three clusters (Fig. 10*A*).

Despite this heterogeneity, SST-INs and VIP-INs still appeared in distinct clusters at hierarchical level 2 of the IN2 subpopulation: 90% of all confirmed VIP-INs were clustered into the left branch, while all confirmed SST-INs were in the right branch (Fig. 11*A*). Similarly, VIP-IN and SST-IN subtypes were largely distinct based on their first three PCs (Fig. 11*F*), indicating some separation of all three IN subtypes. Indeed, both t-SNE (Fig. 11*G*) and LLE (Fig. 11*H*) analyses distinguished subclusters enriched in PV-INs, SST-INs, or VIP-INs, indicating a correlation between intrinsic electrical properties and IN marker gene expression.

To differentiate these three IN types quantitatively, we compared the means and medians for the intrinsic electrical properties of IHC-defined PV-INs, SST-INs, and VIP-INs (Table 6). PV-INs and SST-INs differed significantly in 46 out of their 63 properties, while PV-IN and VIP-IN differed in 53/63 of their properties and SST-INs and VIP-INs differed significantly in 30/63 of their properties (Table 7). To visualize such comparisons, mean Z-scores for all 63 properties are compared for all three IN types in Figure 12. This analysis readily distinguished the intrinsic properties of PV-INs, SST-INs, and VIP-INs. In general, the properties of PV-INs and VIP-INs overlapped least and were often at opposite extremes. SST-INs substantially overlapped with both VIP-INs and PV-INs, with properties typically intermediate to those of PV-INs and VIP-INs and closer to the mean values for the total IN population (Fig. 12, black circle). VIP-INs were the most excitable, with lowest *ct*, most depolarized RMP and highest Rm. PV-INs were the least excitable, with the most hyperpolarized RMP, lowest Rm and highest *ct*, as well as having the shortest AP half-width and highest AP frequency. Characteristic features of SST-INs were relatively low amounts of adaptation and regular AP firing in response to all three stimulus protocols. In summary, the intrinsic electrical properties of three IHC-defined IN subtypes showed significant differences that could be used to identify these IN subtypes.

**Table 6:** Comparison of all 63 cell properties of IHC-confirmed PV-INs, SST-INs, and VIP-INs recorded at the current threshold (ct), twice current threshold (2*ct:), and the depolarizing current that evoked maximum firing frequency (max AP)

Group		PV IN				SST IN				VIP IN			
#	Property	Mean	SEM	Median	MAD	Mean	SEM	Median	MAD	Mean	SEM	Median	MAD
1	RMP (mV)	–66.4	1.20	–67.2	2.30	–62.2	1.10	–63.0	4.10	–59.8	0.90	–59.8	2.50
2	Rm (MΩ)	159	10.5	157	29.3	414	31.0	389	108	653	38.7	616	148
3	current threshold (ct: pA)	165	11.0	160	40.0	54.3	6.40	40.0	20.0	24.0	1.90	20.0	0.00
4	ct: AHP amplitude (mV)	–22.8	0.90	–22.4	2.00	–21.2	1.00	–20.0	3.50	–14.3	0.70	–13.4	2.40
5	ct: AHP latency (ms)	1.88	0.09	1.80	0.28	4.91	1.11	3.58	0.46	4.30	0.17	4.17	0.56
6	ct: max AP rise (mV/ms)	243	11.5	263	17.2	180	7.10	184	28.1	161	6.60	154	32.0
7	ct: max AP decay (mV/ms)	–127	6.40	–134	21.9	–65.0	2.70	–66.4	9.90	–47.6	1.70	–45.3	4.70
8	ct: AP half-width (ms)	0.71	0.03	0.67	0.09	1.20	0.04	1.19	0.09	1.60	0.04	1.59	0.12
9	ct: AP threshold (mV)	–37.5	1.20	–37.2	2.60	–33.4	0.70	–34.2	2.80	–33.3	0.70	–33.4	2.30
10	ct: AP amplitude (mV)	67.6	1.70	68.3	4.00	69.5	1.60	70.6	6.60	72.4	1.00	72.4	4.10
11	ct: AP rise/decay ratio	1.92	0.04	1.86	0.11	2.83	0.12	2.79	0.41	3.42	0.12	3.25	0.36
12	ct: AP threshold/AP amp.	–0.55	0.01	–0.56	0.03	–0.48	0.01	–0.49	0.02	–0.46	0.01	–0.47	0.03
13	ct: AP rise/ AP half-width	362	27.7	395	90.8	158	9.70	162	29.3	105	6.80	99.2	23.0
14	2xct: # AP	67.3	4.40	71.0	13.0	22.5	2.30	20.5	7.00	14.9	1.00	14.5	3.50
15	2xct: latency to first AP (ms)	7.70	0.80	7.70	1.40	36.4	4.60	31.3	14.0	37.3	4.40	33.4	14.0
16	2xct: AHP amplitude (mV)	–17.0	0.80	–16.7	2.60	–18.6	0.90	–17.9	3.80	–13.4	0.80	–13.3	2.30
17	2xct: AHP latency	1.80	0.07	1.78	0.24	3.33	0.16	3.10	0.44	4.00	0.12	4.03	0.36
18	2xct: max AP rise (mV/ms)	253	10.7	270	30.5	182	8.60	187	21.1	160	6.00	159	27.3
19	2xct: max AP decay (mV/ms)	–125	5.20	–130	12.8	–67.0	2.90	–67.9	7.10	–49.2	1.50	–49.4	4.70
20	2xct: AP half-width (ms)	0.77	0.03	0.73	0.10	1.16	0.04	1.13	0.10	1.55	0.04	1.53	0.12
21	2xct: AP threshold (mV)	–41.6	1.00	–43.1	2.50	–34.7	0.70	–35.2	1.50	–34.3	0.70	–34.4	1.90
22	2xct: AP amplitude (mV)	70.3	1.40	70.9	2.00	69.6	1.50	70.6	5.50	72.3	1.00	72.1	3.30
23	2xct: AP rise/decay ratio	2.00	0.00	2.00	0.10	2.80	0.10	2.70	0.30	3.30	0.10	3.20	0.30
24	2xct: AP thresh/AP ampl	–0.59	0.01	–0.61	0.02	–0.50	0.01	–0.51	0.02	–0.47	0.01	–0.48	0.04
25	2xct: AP rise/ AP half-width	345	23.0	372	63.5	168	13.2	166	43.1	108	6.20	103	21.9
26	2xct: amp 1st/2nd AP	1.05	0.01	1.05	0.02	1.06	0.01	1.04	0.02	1.11	0.01	1.09	0.05
27	2xct: amp 2nd/3rd AP	1.01	0.00	1.01	0.01	1.02	0.00	1.02	0.01	1.02	0.01	1.01	0.01
28	2xct: amp 1st /last AP	1.13	0.02	1.13	0.05	1.15	0.02	1.12	0.06	1.26	0.04	1.19	0.09
29	2xct: initial instant frequency (Hz)	92.7	5.30	86.0	18.1	33.0	3.20	30.2	9.00	32.5	2.30	31.4	8.70
30	2xct: adaptation (Hz)	25.4	2.90	24.0	9.30	10.9	1.70	10.4	4.30	17.7	2.70	19.2	9.70
31	2xct: ISI ratio	0.73	0.03	0.74	0.05	0.69	0.03	0.70	0.09	0.49	0.06	0.44	0.19
32	2xct: mean of first 2 ISI (ms)	12.2	0.80	11.9	2.00	43.7	5.10	34.9	10.7	38.2	2.50	34.3	6.80
33	2xct: SD of first 2 ISI (ms)	1.00	0.10	1.00	0.50	4.80	1.10	3.00	1.70	7.60	1.30	5.50	4.00
34	2xct: change of ISI durationfrom 1st to 2nd AP (ms)	1.50	0.20	1.40	0.70	6.80	1.60	4.30	2.40	3.90	2.60	6.40	6.10
35	2xct: change of instant frequencyfrom 1st to 2nd AP pair (Hz)	–10.6	1.20	–10.4	3.70	–5.00	1.10	–4.00	2.10	–4.80	2.00	–6.70	5.30
36	2xct: Cv2:all	0.05	0.02	0.03	0.01	0.08	0.01	0.06	0.02	0.23	0.03	0.21	0.11
37	2xct: Cv2-1st AP	0.05	0.02	0.03	0.01	0.07	0.01	0.05	0.02	0.23	0.03	0.16	0.10
38	2xct: Cv2-1st/2nd AP	0.05	0.02	0.03	0.01	0.07	0.01	0.04	0.02	0.23	0.03	0.17	0.11
39	Max AP: # AP	93.4	5.20	97.0	14.0	37.3	2.30	37.0	9.50	23.4	1.50	22.5	6.50
40	Max AP: latency to first AP (ms)	4.00	0.40	3.10	0.80	11.2	0.90	11.6	3.50	21.7	4.10	13.4	4.70
41	Max AP: AHP amplitude (mV)	–12.7	0.80	–13.4	3.40	–15.4	0.90	–15.3	3.60	–11.4	0.90	–11.4	3.00
42	Max AP: AHP latency (ms)	1.77	0.07	1.73	0.29	2.90	0.12	2.78	0.33	3.75	0.12	3.73	0.41
43	Max AP: max AP rise (mV/ms)	269	14.6	277	25.4	178	10.1	178	26.1	158	6.70	152	28.9
44	Max AP: max AP decay (mV/ms)	–127	6.80	–127	19.5	–66.6	3.30	–66.4	7.00	–48.9	1.90	–46.9	3.90
45	Max AP: AP half-width (ms)	0.84	0.03	0.81	0.09	1.14	0.04	1.16	0.13	1.54	0.04	1.51	0.11
46	Max AP: AP threshold (mV)	–43.4	1.40	–44.3	4.30	–34.7	0.80	–35.1	1.60	–33.8	0.60	–34.0	2.00
47	Max AP: AP amplitude (mV)	70.0	1.40	70.8	2.40	67.5	1.40	67.7	4.80	70.7	0.80	70.7	3.10
48	Max AP: AP rise/decay ratio	2.12	0.04	2.06	0.11	2.68	0.07	2.71	0.27	3.25	0.09	3.23	0.32
49	Max AP: AP thresh/AP amp	–0.62	0.01	–0.64	0.02	–0.51	0.01	–0.51	0.02	–0.48	0.01	–0.48	0.04
50	Max AP: AP rise/AP half-width	335	26.5	335	64.8	166	15.1	158	38.2	107	7.20	100	21.0
51	Max AP: amp 1st/2nd AP	1.11	0.00	1.11	0.02	1.14	0.02	1.12	0.04	1.22	0.02	1.22	0.12
52	Max AP: amp 2nd/3rd AP	1.04	0.00	1.04	0.01	1.04	0.01	1.03	0.01	1.05	0.01	1.05	0.03
53	Max AP: amp 1st /last AP	1.37	0.03	1.35	0.10	1.42	0.03	1.41	0.09	1.48	0.04	1.48	0.18
54	Max AP: initial instant frequency (Hz)	139	6.30	140	17.1	71.7	4.20	71.0	15.8	58.1	4.70	57.9	24.5
55	Max AP: adaptation (Hz)	50.2	2.80	48.9	8.80	38.3	3.20	37.0	10.9	36.5	4.50	38.1	19.5
56	Max AP: ISI ratio	0.60	0.00	0.70	0.00	0.50	0.00	0.50	0.10	0.50	0.10	0.30	0.10
57	Max AP: Mean of first 2 ISI (ms)	8.10	0.50	7.70	0.90	16.6	1.10	15.2	3.20	26.3	2.80	21.4	7.70
58	Max AP:SD of first 2 ISI (ms)	0.70	0.10	0.60	0.10	1.70	0.20	1.50	0.70	6.00	1.50	3.60	1.70
59	Max AP: change of ISI durationfrom 1st to 2nd AP (ms)	1.00	0.10	0.90	0.20	2.10	0.30	2.00	0.90	5.70	2.40	4.30	2.70
60	Max AP: change of instantaneous frequency from 1st to 2nd AP pair (Hz)	–15.6	1.10	–16.2	3.60	–9.80	1.30	–9.20	3.60	–14.9	2.80	–16.2	6.70
61	Max AP: Cv2:all	0.03	0.00	0.03	0.01	0.07	0.01	0.06	0.02	0.18	0.03	0.11	0.05
62	Max AP: Cv2-1st AP	0.03	0.00	0.02	0.01	0.07	0.01	0.06	0.02	0.17	0.03	0.10	0.05
63	Max AP: Cv2-1st/2nd AP	0.03	0.00	0.02	0.01	0.07	0.01	0.05	0.02	0.17	0.03	0.10	0.05

Cell property values are given as means and their SEM and medians and their median absolute deviation (MAD). Cell features are sorted according to the radar plots in Figure 12.

**Table 7.** Statistical comparison of the intrinsic cell features of all IHC-confirmed PV-INs, SST-INs, and VIP-INs

Statistical test	# cell property	Cell property	ANOVA			Tukey’s *posthoc t* test		
			F_(2,76)_	*p* value	*R*^2^	PV vs SST	PV vs VIP	SST vs VIP
ANOVA and Tukey’s*post hoc t* test	1	RMP (mV)	9.00	3.2E-04	0.20	2.5E-02	1.9E-04	2.1E-01
	4	ct: AHP amplitude (mV)	28.1	8.3E-10	0.43	4.6E-01	2.0E-08	1.8E-07
	9	ct: AP threshold (mV)	7.76	8.8E-04	0.17	2.6E-03	1.7E-03	9.9E-01
	10	ct: AP amplitude (mV)	2.75	7.0E-02	0.07	6.4E-01	6.6E-02	2.9E-01
	12	ct: AP threshold/AP amp	28.1	8.2E-10	0.43	1.5E-06	5.7E-09	1.2E-01
	16	2xct: AHP amplitude (mV)	11.4	5.0E-05	0.23	4.3E-01	1.3E-02	4.0E-05
	17	2xct: AHP latency (ms)	62.4	1.3E-16	0.63	5.3E-09	5.1E-09	8.8E-04
	20	2xct: AP half-width (ms)	81.5	2.0E-19	0.69	7.5E-08	5.1E-09	7.6E-09
	21	2xct: AP threshold (mV)	25.2	4.6E-09	0.40	1.4E-07	2.3E-08	8.9E-01
	24	2xct: AP thresh/AP ampl	44.2	2.3E-13	0.54	7.7E-09	5.1E-09	5.6E-02
	41	Max AP: AHP amplitude (mV)	5.70	5.0E-03	0.13	1.1E-01	6.2E-01	3.9E-03
	42	Max AP: AHP latency (ms)	70.6	7.1E-18	0.66	1.9E-08	5.1E-09	6.2E-07
	53	Max AP: amp 1st /last AP	2.97	5.7E-02	0.07	5.5E-01	5.1E-02	3.1E-01
	55	Max AP: adaptation (Hz)	3.18	4.8E-02	0.08	1.1E-01	4.9E-02	9.3E-01
Statistical test	# cell property	Cell property	Kruskal–Wallistest			Dunn’s multiplecomparison		
			*H* value	*p* value	η2	PV vs SST	PV vs VIP	SST vs VIP
	2	Rm (MΩ)	51.8	5.8E-12	0.68	7.4E-05	1.9E-12	3.4E-03
	3	ct (pA)	53.5	2.4E-12	0.70	6.8E-05	8.0E-13	2.3E-03
	5	ct: AHP latency (ms)	43.9	2.9E-10	0.58	1.2E-06	3.1E-10	4.3E-01
	6	ct: max AP rise (mV/ms)	25.5	2.9E-06	0.34	1.1E-03	1.7E-06	3.6E-01
Kruskal–Wallis:Dunn's multiplecomparison test	7	ct: max AP decay (mV/ms)	54.2	1.7E-12	0.71	2.0E-04	6.5E-13	7.2E-04
	8	ct: AP half-width (ms)	56.6	5.1E-13	0.74	3.8E-04	2.3E-13	1.8E-04
	11	ct: AP rise/decay ratio	45.8	1.2E-10	0.60	6.1E-05	4.2E-11	2.0E-02
	13	ct: AP rise/AP half-width	47.2	5.6E-11	0.62	2.8E-04	2.0E-11	3.6E-03
	14	2xct: # AP	44.0	2.8E-10	0.58	8.9E-06	1.4E-10	1.2E-01
	15	2xct: latency to first AP	38.9	3.5E-09	0.51	2.6E-07	2.1E-08	1.0E+00
	18	2xct: max AP rise (mV/ms)	29.4	4.2E-07	0.39	5.6E-04	2.2E-07	2.3E-01
	19	2xct: max AP decay (mV/ms)	52.7	3.7E-12	0.69	1.3E-04	1.3E-12	1.6E-03
	22	2xct: AP amplitude (mV)	1.23	5.4E-01	0.02	1.0E+00	1.0E+00	1.0E+00
	23	2xct: AP rise/decay ratio	46.0	1.0E-10	0.61	1.1E-04	3.5E-11	1.1E-02
	25	2xct: AP rise/AP half-width	43.8	3.1E-10	0.58	5.9E-04	1.1E-10	4.6E-03
	26	2xct: amp 1st/2nd AP	17.6	1.5E-04	0.23	1.0E+00	1.3E-02	1.9E-04
	27	2xct: amp 2nd/3rd AP	2.20	3.3E-01	0.03	1.0E+00	1.0E+00	1.0E+00
	28	2xct: amp 1st /last AP	7.31	2.6E-02	0.10	1.0E+00	6.9E-02	6.4E-02
	29	2xct: initial instantaneous frequency	40.1	1.9E-09	0.53	3.6E-08	4.9E-08	1.0E+00
	30	2xct: adaptation (Hz)	15.8	3.7E-04	0.21	2.2E-04	1.0E-01	1.0E-01
	31	2xct: ISI ratio	19.2	6.7E-05	0.25	1.0E+00	2.5E-04	1.7E-03
	32	2xct: mean of first 2 ISI (ms)	41.2	1.2E-09	0.54	3.7E-08	2.0E-08	1.0E+00
	33	2xct: SD of first 2 ISI (ms)	23.2	9.1E-06	0.31	3.5E-03	4.8E-06	2.8E-01
	34	2xct: change of ISI duration from 1st to 2nd AP (ms)	10.6	5.0E-03	0.14	7.3E-03	1.7E-02	1.0E+00
	35	2xct: change of instant frequency from 1st to 2nd AP pair (Hz)	10.7	4.8E-03	0.14	4.3E-03	3.7E-02	1.0E+00
	36	2xct: Cv2:all	34.5	3.2E-08	0.45	1.7E-01	7.0E-08	1.4E-04
Kruskal–Wallis: Dunn's multiplecomparison test	37	2xct: Cv2-1st AP	35.0	2.5E-08	0.46	1.8E-01	6.1E-08	1.1E-04
	38	2xct: Cv2-1st/2nd AP	35.3	2.1E-08	0.46	2.3E-01	6.7E-08	6.9E-05
	39	Max AP: # AP	52.5	3.9E-12	0.69	7.9E-05	1.3E-12	2.6E-03
	40	Max AP: Latency to first AP	40.8	1.4E-09	0.54	1.6E-05	7.6E-10	1.7E-01
	43	Max AP: max AP rise (mV/ms)	29.9	3.2E-07	0.39	2.2E-04	2.0E-07	3.7E-01
	44	max AP: max AP decay (mV/ms)	52.1	4.8E-12	0.69	2.7E-04	1.8E-12	9.5E-04
	45	Max AP: AP half-width (ms)	55.2	1.0E-12	0.73	3.1E-03	9.0E-13	2.9E-05
	46	Max AP: AP threshold (mV)	29.7	3.5E-07	0.39	5.4E-05	4.0E-07	9.0E-01
	47	Max AP: AP amplitude (mV)	2.53	2.8E-01	0.03	1.0E+00	1.0E+00	1.0E+00
	48	Max AP: AP rise/decay ratio	48.2	3.3E-11	0.63	6.0E-04	1.3E-11	1.3E-03
	49	Max AP: AP thresh/AP ampl	39.0	3.4E-09	0.51	1.8E-04	1.4E-09	4.6E-02
	50	Max AP: AP rise/ AP half-width	42.7	5.3E-10	0.56	4.6E-04	2.0E-10	7.9E-03
	51	Max AP: 1st/2nd AP ratio	10.4	5.6E-03	0.14	8.1E-01	6.2E-03	8.5E-02
	52	Max AP: 2nd/3rd AP ratio	1.79	4.1E-01	0.02	1.0E+00	1.0E+00	1.0E+00
	54	Max AP: initial instantaneous frequency	38.8	3.7E-09	0.51	5.8E-06	3.6E-09	4.9E-01
	56	Max AP: ISI ratio	18.3	1.1E-04	0.24	5.5E-03	7.3E-05	7.1E-01
	57	Max AP: Mean of first 2 ISI (ms)	42.9	4.7E-10	0.57	2.9E-05	2.0E-10	6.8E-02
	58	Max AP: SD of first 2 ISI (ms)	49.2	2.1E-11	0.65	2.9E-03	1.3E-11	2.0E-04
	59	Max AP: change of ISI duration from 1st to 2nd AP (ms)	25.0	3.8E-06	0.33	1.3E-02	1.8E-06	5.9E-02
	60	Max AP: change of instant frequency from 1st to 2nd AP pair (Hz)	8.02	1.8E-02	0.11	2.0E-02	9.8E-01	1.5E-01
	61	Max AP: Cv2:all	42.0	7.5E-10	0.55	7.9E-04	2.9E-10	5.9E-03
	62	Max AP: Cv2-1st AP	39.7	2.4E-09	0.52	6.9E-04	9.1E-10	1.3E-02
	63	Max AP: Cv2-1st/2nd AP	38.5	4.3E-09	0.51	7.2E-04	1.6E-09	1.7E-02
	Sum of significant difference cell properties					46	53	30

If a cell property showed a normal distribution, ANOVA and Tukey’s *post hoc t* test was used to compare group means. If a cell property showed a non-normal distribution, a Kruskal–Wallis and Dunn’s multiple comparison test was used to compare group medians.

To enhance our ability to distinguish IN subtypes, an artificial neural network was trained with the 63 intrinsic electrical properties of 77 confirmed INs (19 PV-INs, 28 SST-INs, and 30 VIP-INs). Overall, the cross-validation prediction accuracy for distinguishing IN subtypes via this approach was 93%, indicating reliable resolution of the three subtypes of claustrum INs.

In conclusion, measurements of intrinsic electric properties allowed us to distinguish claustral PNs from INs and to resolve subtypes of both PNs and INs. The anatomical projections and expression of marker genes in these neurons largely respected the electrophysiological distinctions between cell types.

### Effects of temperature

Because the recordings described above were done at room temperature (24°C), we also asked whether our classification scheme could be extrapolated to other conditions by determining how warmer temperatures affected cell electrical properties. A representative example of the activity of a PN3 neuron recorded at two different temperatures (24°C and 30°C) is shown in Figure 13*A*. It is evident that the warming the temperature affected the electrical properties of this neuron, for example, altering AP frequency and the amount of current required to evoke APs. For all 13 neurons examined at these two temperatures, we quantified the temperature sensitivity of their intrinsic electrical properties by calculating the temperature coefficient (Q_10_), a measure of how much each property changes for a ten degree rise in temperature:

(1) $${\text{Q}}_{10}={\left({\text{P}}_{\text{2}}/{\text{P}}_{\text{1}}\right)}^{\text{1}0/\left(\text{T2}-\text{T1}\right)}\text{,}$$

where P_2_ is the value of each parameter at temperature T_2_, and P_1_ is the same parameter at a lower temperature, T_1_. A Q_10_ > 1 indicates a parameter that increases with temperature, while values below one indicate parameters that decrease with temperature (Janssen, 1992; Tang et al., 2010).

Most of the intrinsic electrical properties of claustral neurons had Q_10_ values ranging from 0.25 to 4 (Fig. 13*B*; Table 8). Exceptions were the Q_10_ values of some ratiometric parameters, which were extreme due to the effects of ratioing the Q_10_ values of single parameters. To determine whether these Q_10_ values could be used to adapt our cell classification scheme to different temperatures, we examined the ability of the classification scheme to correctly predict the subtypes of the 13 neurons that were examined at the two temperatures (Fig. 13*C*). At 30°C, the scheme often misclassified neurons as strongly-adapting (SA) PN3 or IN subtypes because of the higher AP rise and decay times and faster frequency adaptation at the higher temperature (Fig. 13*C*, center). However, after using the mean Q_10_ values shown in Figure 13*B* to adjust the cellular properties measured at 30°C, cell type identity was predicted with high fidelity: 100% of the neurons recorded at 30°C were correctly predicted to be either PNs or INs (Fig. 13*C*, right). This temperature correction was also 100% accurate at identifying the three IN types and in separating subcortical PNs from cortical PNs. When separating all neurons into their previously defined cell types, the accuracy was reduced to a still acceptable 85%, due to some errors in distinguishing subtypes of cortical PNs.

**Table 8:** Mean and SEM of property-specific Q_10_ ratios

Cell property	Mean Q_10_	SEM
Current threshold (*ct*:pA)	1.67	0.24
RMP (mV)	1.04	0.02
Rm (MΩ)	0.81	0.09
2xct: AP rise/AP half-width	7.75	0.93
ct: AP rise/AP half-width	7.33	0.73
Max AP: AP rise/AP half-width	6.96	1.01
2xct: adaptation (Hz)	3.28	0.68
2xct: max AP decay (mV/ms)	3.27	0.22
Max AP: max AP decay (mV/ms)	3.15	0.30
ct: max AP decay (mV/ms)	2.92	0.18
2xct: max AP rise (mV/ms)	1.80	0.14
ct: max AP rise (mV/ms)	1.75	0.10
Max AP: max AP rise (mV/ms)	1.67	0.16
ct: AP rise/decay ratio	0.62	0.04
2xct: AP rise/decay ratio	0.56	0.03
Max AP: AP rise/decay ratio	0.54	0.03
Max AP: AP half-width (ms)	0.27	0.02
ct: AP half-width (ms)	0.26	0.02
2xct: AP half-width (ms)	0.26	0.02
Max AP: # AP	5.26	1.51
Initial adaptation change (2) (Hz/pA)	5.11	0.99
Max AP: change of instant frequency from 1st to 2nd AP pair (Hz)	4.68	1.63
2xct: initial instant frequency (Hz)	4.60	1.07
2xct: change of instant frequency from 1st to 2nd AP pair (Hz)	4.09	0.75
Max AP: adaptation (Hz)	4.08	1.32
2xct: # AP	4.05	0.74
Max initial adaptation change (Hz/pA)	3.29	0.51
Max AP: initial instant frequency (Hz)	3.28	0.55
2xct: change of ISI duration from 1st to 2nd AP (ms)	1.52	0.44
2xct: SD of first 2 ISI (ms)	1.42	0.42
Max AP: ISI ratio	1.21	0.25
2xct: ISI ratio	1.15	0.15
Max adaptation change relative to ct	1.12	0.14
Max adaptation change relative to ct (2)	1.04	0.15
2xct: mean of first 2 ISI (ms)	0.61	0.15
Max AP: Change of ISI duration from 1st to 2nd AP (ms)	0.53	0.30
Max AP: Mean of first 2 ISI (ms)	0.41	0.10
Max AP:SD of first 2 ISI (ms)	0.20	0.09
2xct: AP thresh/AP amp	1.72	0.11
ct: AP thresh/AP amp	1.69	0.10
Max AP: AP thresh/AP amp	1.67	0.11
ct: AHP amplitude (mV)	1.18	0.19
Max AP: AHP amplitude (mV)	1.09	0.33
ct: AP threshold (mV)	1.05	0.03
Max AP: AP threshold (mV)	1.03	0.05
2xct: AP threshold (mV)	1.00	0.03
2xct: amp 2nd/3rd AP	0.98	0.03
Max AP: amp 2nd/3rd AP	0.90	0.04
2xct: AHP amplitude (mV)	0.85	0.17
2xct: amp 1st /last AP	0.76	0.03
Max AP: amp 1st /last AP	0.75	0.06
Max AP: amp 1st/2nd AP	0.73	0.03
2xct: amp 1st/2nd AP	0.69	0.03
ct: AP amplitude (mV)	0.64	0.03
Max AP: AP amplitude (mV)	0.64	0.04
2xct: AP amplitude (mV)	0.62	0.03
Mean ADP value (mV)	0.87	0.29
ADP probability	0.84	0.08
ct: AHP latency (ms)	0.67	0.15
2xct: Cv2:all	0.66	0.10
2xct: latency to first AP (ms)	0.64	0.12
2xct: Cv2-1st/2nd AP	0.62	0.08
2xct: Cv2-1st AP	0.61	0.07
Max AP: Cv2-1st/2nd AP	0.60	0.17
Max AP: Cv2-1st AP	0.59	0.16
max AP: latency to first AP (ms)	0.51	0.08
Max AP: Cv2:all	0.51	0.14
2xct: AHP latency	0.26	0.02
Max AP: AHP latency (ms)	0.25	0.02

Q_10_ values were calculated from recordings done at 24°C and 30°C.

In summary, because the intrinsic electrical properties of claustrum neurons scaled predictably with temperature, our classification scheme could accurately recognize different subtypes of neurons at temperatures other than room temperature.

### Neural network assisted claustral cell classification

**Figure 14.**
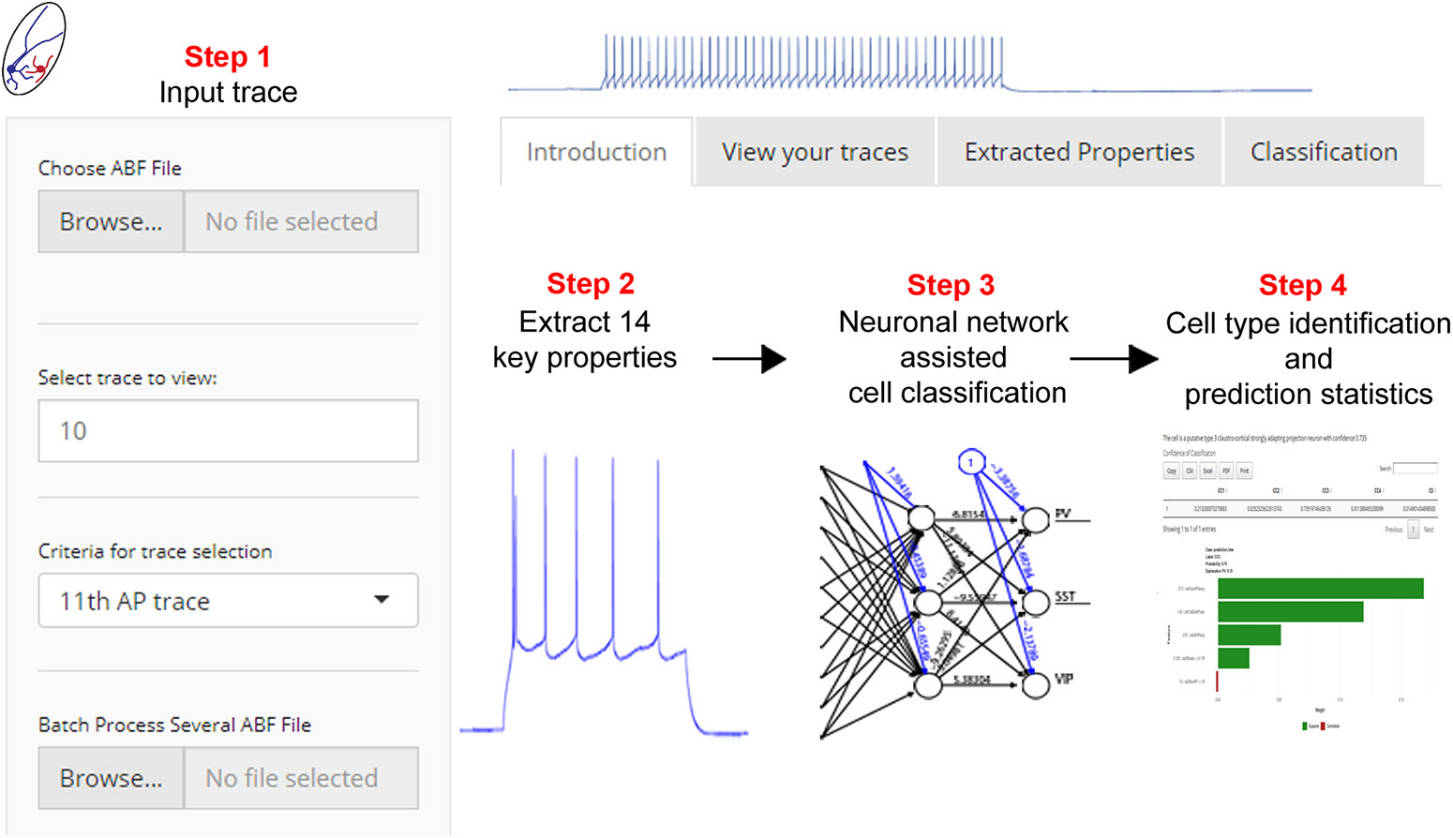
The ClaustrumClassifier (ClaCla): a neural-network assisted cell classification tool. A trained neural network developed to facilitate the classification of claustral neurons in four steps. Step 1, Input raw data file. Step 2, Automated extraction of intrinsic electrical properties. Step 3, Neuronal network assisted cell classification. Step 4, The classifier returns the likelihood that the classified cell belongs to one of the eight cell subtypes.

While intrinsic electrical properties can unambiguously identify subtypes of claustral neurons, to achieve high accuracy it is necessary to consider multiple cellular properties, particularly for distinguishing IN subtypes. To simplify the process of identifying claustrum neurons according to their intrinsic electrical properties, we trained a neural network to classify claustral cells (Fig. 14) using the open-source programming language R (R-Core-Team, 2018). The tool analyzes experimental recordings saved in the .abf format (Fig. 14, step 1) and extracts 14 key intrinsic electrical properties from raw data (Fig. 14, step 2). The extracted properties are then automatically compared with our database of five PN and three IN subtypes, and the network-assisted cell classification procedure (Fig. 14, step 3) defines the probability that a neuron is one of the eight cell subtypes (Fig. 14, step 4). Tests of the classifier revealed that its prediction accuracy for initially identifying a neuron as IN versus PN was 97%, for the identification of PN subtypes was 89%, and for IN subtypes the classifier had a prediction accuracy of 93%. In summary, the classifier provides a user-friendly approach that automatically extracts relevant raw data that are then used to classify claustral neurons. This tool is freely available at https://claustrum.shinyapps.io/classifier/ or https://github.com/adityanairneuro/claustrum.

## Discussion

We have described an electrophysiology-based scheme for classifying claustral neurons that was augmented by anatomical tracer studies and analyses of marker gene expression. Five subtypes of claustral PNs could be distinguished, with PN subtypes that predominantly projected to either cortical or subcortical targets, as well as three subtypes of INs that expressed PV, SST, or VIP. In addition, we developed a cell classifier tool that facilitates data extraction and identification of claustral neurons according to their intrinsic electrical properties.

### Intrinsic electrical properties of claustral neurons

Our work substantially extends previous efforts to characterize the intrinsic electrical properties of claustrum neurons.

#### PNs

Kim et al. (2016) described a few of the intrinsic electrical properties of claustro-cortical PNs, identified via retrograde labeling, that exhibited variable degrees of AP frequency adaptation. Although they did not attempt to distinguish PN subtypes, their results likely correspond to our PN subtypes 2–4. Shibuya and Yamamoto (1998) identified two distinct neuron types in rat claustrum that differed in their frequency adaptation and called these “slow-adapting neurons” and “fast-adapting neurons.” Both of these resemble our subtypes of claustro-cortical PN: their slow-adapting neurons likely correspond to our cortical-projecting type PN2 or PN3, while the fast-adapting neurons most closely resemble PN4 and PN5. Our results also improve on the classification scheme of Chia et al. (2017) that distinguished strongly-adapting (SA) and mildly-adapting (MA) populations of anterior cingulate cortex-projecting claustral neurons. Both of our approaches agree that the claustrum contains multiple subtypes of PNs that can be distinguished based on their AP frequency adaptation: the SA neurons of Chia et al. (2017) likely correspond to PN3–5, while the MA neurons likely correspond to our cortical-projecting PN2 subtype, which only show adaptation at relatively high levels of depolarization.

Chia et al. (2017) also reported a sex-specific enrichment of distinct PN subtypes, depending on the rostro-caudal location of the PN subtype. Although we observed a trend for certain PN types to be more enriched in males or females, none of those trends were statistically significant, presumably because our analysis did not sort neurons according to their locations. Our classification scheme also extends the results of White and Mathur (2018), who described two types of claustral PN that differed in their intrinsic properties and AP frequency adaptation. Their type 2 neurons are comparable to the vigorous doublet-AP firing PN3 neurons we have described, while their type 1 neurons are likely to be PN2 neurons that show less frequency adaptation and reduced initial doublet spiking. No PN1 subtype neurons have been described previously; this is due to the fact that no previous analyses considered claustral PN projecting to subcortical regions.

It was somewhat surprising to find claustrum neurons that projected to subcortical structures. Previous work has suggested that the primary subcortical target of the claustrum is the amygdala (Zingg et al., 2018), which we did not examine in our experiments. Although injecting retrobeads into the subcortex mainly labeled insular neurons (Mathur et al., 2009), there was also sparse labeling of neurons within the PV-enriched claustral core/shell region and we only recorded from neurons that were clearly within this region. Injection of retrobeads into subcortical regions also yielded some bead leakage into cortical structures along the injection track. However, because our subcortical bead injections almost exclusively labeled PN1 cells, which were not labeled by cortical bead injections, we can deduce that leakage of tracer into the cortex had minimal impact on our experimental results. Thus, we conclude that PN1 cells genuinely project to subcortical structures, such as the mediodorsal thalamus, central medial thalamic nucleus, ventromedial thalamic nucleus, or the habenula (Table 1), and that the function of claustral PN1 neurons is to provide input to these structures.

In summary, our classification scheme largely unifies previous reports and extends our understanding by distinguishing multiple subtypes of claustral PNs that project to different brain areas.

#### INs

Neither Shibuya and Yamamoto (1998) nor Chia et al. (2017) examined claustral INs. Kim et al. (2016) recorded from genetically-defined PV-INs and reported properties that are consistent with our observations. They did not consider other types of INs. White and Mathur (2018) described three classes of claustral IN: one class of PV-negative INs (type 3) whose identity was not defined and two classes of PV-positive neurons (type 4 and 5) with distinct firing patterns and intrinsic features. It is likely that their type 3 neurons include both the SST-INs and VIP-INs that we have characterized, as well as potentially other types of INs. The intrinsic properties of their type 4 PV neurons are comparable to our PV-INs (high AP frequency, low relative adaptation, relatively hyperpolarized RMP). However, the intrinsic properties of their type 5 PV neurons do not coincide with those of our PV-INs or those of Kim et al. (2016), which have more hyperpolarized RMP and deeper AHPs than their type 5 cells. We suspect this discrepancy arises because the Cre-driver mouse line used by White and Mathur (2018) ectopically expresses Cre in non-PV cells, including PNs (Tanahira et al., 2009). Our classifier provides the first reliable way to use intrinsic electrical properties to distinguish claustral PV-INs, SST-INs, and VIP-INs.

In summary, our results characterize many different subtypes of claustrum neurons, including previously unknown PN and IN subtypes, and lay the foundation for future studies of claustrum neurons and their functions. From a technical perspective, our work substantially advances previous efforts to identify types of claustrum neurons by systematically analyzing up to 63 intrinsic electrical properties of claustrum neurons and by integrating these properties with information about projection targets and expression of IN marker genes. Our efforts have also yielded an objective algorithm for classifying claustrum neurons that was validated by multiple non-overlapping approaches. Although our classification scheme was based on recordings done under one set of experimental conditions, at 24°C and in adult mice (postnatal age 65), it is largely applicable under other experimental conditions. Specifically, we found that our classification scheme works at higher temperatures, after taking into account the temperature dependence of intrinsic electrical properties. The characteristic firing patterns of claustrum neurons, such as strong frequency adaptation with an initial AP doublet or amplitude adaptation with mild frequency adaptation, are also observed in neurons from younger age mice (mean age: postnatal age 21 ± 0.4; unpublished results of Y. Tang and M. Graf), and we anticipate that our classification scheme will apply over a range of ages. Hence, our classification rules can be used under a variety of experimental conditions.

### Comparison of claustral and cortical neurons

In cerebral cortex, INs differ fundamentally from PNs (McCormick et al., 1985; Gouwens et al., 2019) and can be grouped into three main subtypes: PV-INs, SST-INs, and 5Ht3aR-INs, with the latter further subdivided into VIP-expressing and non-expressing INs (Lee et al., 2010; Rudy et al., 2011). Although the intrinsic properties of different cortical IN types are variable, certain combinations of properties are enriched in distinct IN types and can be used to cluster them with unsupervised methods (Gouwens et al., 2019). As in the cortex, our trained neural network was able to identify rules that could be used to distinguish fast-spiking PV-INs from irregular-firing VIP-INs and regular-firing SST-INs.

How do the intrinsic electrical properties of neurons in the subcortical claustrum compare to those of cortical neurons? Overall, claustral neurons show trends very similar to those of cortical cells. As in the cortex, the intrinsic features of claustral PNs correlate with differences in their projection targets. For example, there are pronounced differences in intrinsic electrical properties between intratelencephalic PNs that connect to cortical areas (and the striatum) and extratelencephalic PNs that additionally project to areas outside the telencephalon such as brain stem or thalamus (Baker et al., 2018). Similarly, subcortical projecting claustral neurons (PN1) showed less frequency adaptation than neurons that connect to the cortex (PN2–PN4) and PN5. Cortical PN that target different areas also differ in their AP threshold (for review, see Baker et al., 2018). Claustro-cortical PN subtypes differed in some of their electrical properties, suggesting that these subtypes may differ in their cortical projections. Future studies using refined retrograde labeling procedures will be required to consider this possibility. Claustral PV-INs, SST-INs, and VIP-INs generally are comparable to their cortical counterparts and differences between these IN subtypes are consistent between claustral and cortical INs, with PV-INs and VIP-INs differing the most and SST-INs intermediate.

The high similarity of claustral and cortical neurons could arise from their similar developmental origins (Watson and Puelles, 2017; Binks et al., 2019): both cortical and claustral PNs are of pallial origin, while INs originate in subpallial areas and then migrate into cortex or claustrum. As a result of their shared developmental origin, claustral and cortical neurons perhaps could also show similarities in their connectivity patterns.

### New insights into claustrum function

Although our study was intended to lay a cellular foundation for future system-level studies of claustrum function, our work already provides some initial and novel insights into how the claustrum processes information.

#### PNs

AP doublet firing by cortical-projecting claustrum PN2–4 should have significant consequences for information encoding by these cells. Because the depolarization required for initial ADP generation in PN2–PN4 was 1.5 times higher than AP threshold, multiple spiking in these neurons depends non-linearly on input strength. Further, double spiking can cause synaptic facilitation that transiently increases presynaptic release probability (Lisman, 1997). Thus, such doublet-firing properties could introduce non-linearities in transmission to downstream targets. For example, if two separate populations of claustro-cortical PNs receive signals from two different presynaptic inputs, the neurons with weaker input would encode incoming information more linearly and with a lower likelihood of eliciting large postsynaptic potentials, while the supralinear properties associated with double spiking would cause neurons receiving stronger input to have a disproportionately higher probability of transmitting this information to their postsynaptic targets. This could provide a mechanism to differentiate between relevant and irrelevant signals in the claustro-cortical network and might underlie the reported ability of claustro-cortical neurons to serve as a selective attention filter that depends on the strength of their inputs (Mathur, 2014; Remedios et al., 2014; Goll et al., 2015).

In contrast, subcortical projecting PN1 lack AP doublet firing. The resulting reduction in AP frequency adaptation will confer more linear transmission properties on these neurons during initial AP firing. In addition, PN1 AP amplitude often adapts during repetitive firing. Because synaptic transmission is a sensitive function of AP amplitude (Katz and Miledi, 1967), amplitude adaptation would yield a time-dependent reduction in the ability of claustro-subcortical cells to excite their postsynaptic target cells. In summary, the intrinsic properties of PNs could significantly shape claustrum network activity more globally. Our classification scheme provides a way to identify these subpopulations of PNs and, thereby, to design future experiments that determine how differences in their AP firing contribute to claustrum function.

#### INs

Our discovery that each subtype of claustral IN has unique intrinsic electrical properties also suggests potential differences in the function of these INs. In particular, VIP-INs are the most excitable neurons (highest Rm, lowest *ct*, relative depolarized RMP) and therefore could be preferentially engaged in claustral responses to weak excitatory input.

Considering that the activity of VIP neurons is modulated by expectation (Krabbe et al., 2019; Garrett et al., 2020), and that claustral VIP neurons disinhibit PNs (Graf and Augustine, 2019) in a fashion similar to what has been described for cortical areas (Lee et al., 2013; Pfeffer et al., 2013; Pi et al., 2013), the function of VIP-INs might be to increase the responsiveness of claustral PNs to weak stimuli during top-down modulated attention. Thus, in the context of the proposed role of the claustrum in defining salient information from noise (Mathur, 2014; Remedios et al., 2014; Goll et al., 2015), the unique electrical properties of VIP-INs could allow claustral PNs to respond to weak signals that are otherwise insufficient to activate claustral PNs and thereby determine whether or not an incoming signal is attention worthy.

## Conclusions

Our results provide a comprehensive analysis of the intrinsic electrical properties of claustrum neurons and demonstrate that these properties can be used to distinguish different subtypes of claustral neurons. This classification scheme will serve as the foundation for future studies of the cell-type-specific functions of claustrum neurons. For example, our lab is now using it successfully to discover cell-specific differences in the local inhibitory microcircuitry of the claustrum (Graf and Augustine, 2019), cholinergic gain control of PNs (Nair et al., 2019), and serotoninergic modulation of claustral cell activity (Wong and Augustine, 2019). Finally, by analogy to the Blue Brain Project (Markram et al., 2015), this refined understanding of the properties of claustral neuron subtypes will facilitate development of large-scale models of claustrum networks and will enable a better understanding of higher-level signal processing within the claustrum, as well as between the claustrum and its connected structures.

## Synthesis

Reviewing Editor: Upinder S. Bhalla, National Centre for Biological Sciences, Tata Institute of Fundamental Research; Decisions are customarily a result of the Reviewing Editor and the peer reviewers coming together and discussing their recommendations until a consensus is reached. When revisions are invited, a fact-based synthesis statement explaining their decision and outlining what is needed to prepare a revision will be listed below. The following reviewer(s) agreed to reveal their identity: NONE. Note: If this manuscript was transferred from JNeurosci and a decision was made to accept the manuscript without peer review, a brief statement to this effect will instead be what is listed below.

This is a careful and detailed study on claustral neurons and their classification. The dataset is very substantial and the authors have made significant improvements to the manuscript based on some very thorough referee comments.

The authors have recorded from over 300 neurons and used marker expression, projections, and morphology to complement a large number of electrical parameters extraced from patch recordings. The authors have provided a useful online classifier that takes electrical recordings and classifies the likely claustral neuron subtype.

The one additional thing I would ask of the authors is to provide a link to the github or other standard site for the classifier code and model weights dataset.

The paper is in excellent shape for publication and will be a valuable resource for the field.
